# Novel Criteria to Provide a Locality/Normality Degree in Molecules and Their Relevance in Physical Chemistry

**DOI:** 10.3390/molecules29153490

**Published:** 2024-07-25

**Authors:** Eduardo Suárez, Oscar Guzmán-Juárez, Renato Lemus

**Affiliations:** Instituto de Ciencias Nucleares, Universidad Nacional Autónoma de México, A.P. 70-543, Circuito Exterior, C. U., Mexico City 04510, Mexico; easp_24@comunidad.unam.mx (E.S.); 315064973@quimica.unam.mx (O.G.-J.)

**Keywords:** local normal criteria, algebraic approach, Raman spectroscopy, infrared spectroscopy, vibrational spectroscopy

## Abstract

In contrast to the traditional analysis of molecules using local mode behavior, where the degree of locality is given through a function in terms of Morse potential parameters, new criteria for locality/normality (LN) suitable for application to any molecular system are proposed. The approach is based on analysis of the connection between the algebraic normal and local mode representations. It is shown that both descriptions are equivalent as long as the polyad (total number of quanta) in the local representation is not conserved. The constraint of a local polyad conservation naturally provides a criterion for assigning an LN degree in quantitative form, without an analogue in configuration space. The correlation between the different parameters reveals the physical properties of molecules. A clear connection between the LN degree (based on the fundamentals) and spectroscopic properties is also presented, suggesting a promising approach for identifying mixtures of isotopologues.

## 1. Introduction

Vibrational degrees of freedom can be identified either with a local or a normal mode behavior. Until the seventies, the point of view of description in terms of normal modes dominated, due in part to the success in describing spectra through the inclusion of resonances in the Hamiltonian [1,2]. Examples of this success include the descriptions of H_2_O and CO_2_ [3,4]. This situation changed during the eighties with the advent of modern spectroscopy techniques based on lasers [5,6,7], unveiling doublets in the energy spectra of molecules involving bonds with large mass differences, although evidence of such patterns had been identified many years before [8,9,10,11]. This kind of spectra, although difficult to describe in terms of normal modes, were relatively easy to interpret in terms of interacting local oscillators [12,13,14,15,16]. Indeed, the doublets signal the presence of a local mode behavior and they were explained by the simple model of anharmonic oscillators harmonically coupled (AOHC) [17]. Regarding this model, the general features of the spectra are explained in terms of both the anharmonicity and strength of the interaction between the oscillators, as a consequence of the close relation between anharmonicity and locality [18]. It was realized that in every molecule with bonds involving large mass differences, like for instance H_2_O, CH_4_, and AsH_3_, the stretching degrees of freedom cn be well described in terms of interacting Morse oscillators. In contrast, the bending degrees of freedom are still treated in terms of normal modes, due to their collective features [19]. Within a set of molecules presenting a local mode behavior, it is possible to assign a locality degree using the parameter ξ defined by [12,13]

(1) $$\xi =\left|\frac{2}{\pi }\arctan\left(\frac{\lambda }{\omega x}\right)\right|,$$

where ωx stands for the Morse anharmonicity and λ corresponds to the interaction strength of the oscillators. In the local limit (ωx large and λ small) ξ→0, while in the normal limit (ωx small and λ large) ξ→1. This parameter has been calculated for several molecules following the expected behavior, in accordance with the mass ratio ligand/central-atom dictated by the structure of the molecule. In general, the criterion established by the parameter ξ is satisfied for a great variety of molecules. However, when in a series of molecules the anharmonicities are similar and the strengths do not increase in accordance with the mass difference, the local-normal mode behavior leads to unexpected results. This is the case for the series of pnictogen pyramidal hydrides XH_3_, with X = N, P, As, Sb, and Bi. In order to elucidate this type of behavior a new perspective on local-normal behavior was considered [20].

Even for molecules with local mode behavior, a local-normal transition may appear regarding different states of a given multiplet. This situation has been analyzed from a dynamical point of view using methods of non-linear classical mechanics [21,22,23,24,25,26,27,28,29]. Recently, the local to normal mode transition has been studied from a quite different point of view [20,30,31,32]. The basic feature of the proposal is to focus on the problem from a polyad breaking perspective. A molecule with a local mode behavior is characterized by a set of interacting levels associated with a local polyad, defined in terms of local quantum numbers. As long as this polyad is conserved, the molecule maintains a local character. This viewpoint is partial, in the sense that the polyad-conservation depends on the energy. At sufficiently high energy, the local polyad stops being conserved. However, a local behavior may still be present in a wide energy range, as long as the local polyad is conserved. From this perspective, the energy range becomes very narrow for molecules with evident normal mode behavior. This is indeed the case of the CO_2_ molecule, where the central carbon atom is lighter than the ending oxygen atoms. In this situation, it is not possible to define a local polyad. In fact, the concept of a polyad as a pseudo quantum number that embraces the set of interacting states is well defined in a normal mode scheme, and only when this polyad is suitable to be translated into a local scheme is the molecule said to have a local character, otherwise the concept of a local polyad is lost. The question that arises is how to measure the LN degree from this perspective. The traditional parameter (1) is not useful, because it is constrained to molecules with local mode behavior and consequently a new criterion is needed, in order to involve any possible case covering the different molecular systems. On the other hand, this new criterion is not expected to be based on a system of interacting Morse oscillators, because, in principle, molecules with strong normal mode behavior cannot be described starting with local oscillators without breaking the polyad. In this work, we propose a new general different approach based on the analysis of the limit of the normal to the local description. Following this route, it is possible to derive more than one LN criteria. Surprisingly, these criteria include only the analysis of normal modes associated with the fundamentals. In this contribution, we present in detail the analysis of the new LN criteria for two, three, four, and six oscillators. The case of two oscillators has been partially presented in the context of the isotopologues of CO_2_ [33], as well as the three equivalent oscillators in pyramidal molecules [20], but here both are included in detail in the context of an unified treatment that includes any molecular system. We shall show that the proposed parameters are sensitive enough to distinguish any molecule in either the extreme normal or local regimes. The four-oscillator system is included for tetrahedral molecules, to study the situation where spurious states are present, while octahedral molecules involving six oscillators are studied, because several types of interactions are involved. Embracing this set of systems, we consider every situation to test the proposed parameters. We stress that, for the first time, LN parameters are proposed with incidence in molecular properties, such as non-rigidity and resonances, but also in spectroscopy where vibrational excitations are involved.

This paper is organized as follows: The general theory to establish the different LN criteria is presented in Section 2. In Section 3, an analysis involving two oscillators is presented in detail. Section 4 is devoted to the case of three equivalent oscillators. In Section 5, tetra-atomic molecules are analyzed, while in Section 6, the stretching modes of octahedral molecules are studied. Section 7 is devoted to discussing the relation between the parameters proposed and their physical properties. Finally, the conclusions are drawn in Section 8.

## 2. LN Mode Criteria: General Formalism

In this section, we present the general ideas that lead to establishing several independent parameters to measure the LN degree of molecules. We start by considering the situation for an arbitrary number of equivalent oscillators, albeit for the particular case in which the normal coordinates coincide with symmetry-adapted coordinates. This is a common situation in small and medium size molecules, where the energy of the bends are quite different from the stretches. The generalization to normal coordinates involving several coordinates of the same symmetry involves GF formalism and will be discussed in the next section.

The simplest model to describe vibrational degrees of freedom consists in considering the system as a set of independent harmonic oscillators associated with normal coordinates. When normal coordinates span irreducible representations Γ of the symmetry group *G*, the Hamiltonian in terms of bosonic operators takes the form [34]

(2) $$\begin{array}{ccc} {\hat{H}}_{N} & = & \sum_{\Gamma }\frac{ћ\Omega_{\Gamma }}{2}\sum_{\gamma }\left({\hat{A}}_{\Gamma \gamma }^{†}{\hat{A}}_{\Gamma \gamma }+{\hat{A}}_{\Gamma \gamma }{\hat{A}}_{\Gamma \gamma }^{†}\right), \end{array}$$

where the sum over Γ runs over all irreducible representations contained in the reducible representation spanned by the equivalent local oscillators. The subscript index *N* in the Hamiltonian emphasizes the normal mode representation. From the fundamental energies and the explicit expression of the frequencies $\Omega_{\Gamma }=\sqrt{G_{\Gamma \Gamma }^{\left(0\right)}F_{\Gamma \Gamma }}$, we are able to estimate the force constants for both normal and local mode schemes

(3) $$\left\{F_{\Gamma \Gamma }\right\}\;\to \;\left\{f_{q_{i}q_{j}}^{\left(N\right)}\right\}.$$

Here, $G_{\Gamma \Gamma }^{\left(0\right)}$ and $F_{\Gamma \Gamma }$ correspond to the elements of the Wilson matrix and force constants, respectively, in the normal basis. The local force constants $f_{q_{i}q_{j}}^{\left(N\right)}$ associated with the local coordinates $\left\{q_{i}\right\}$ are obtained from the force constants $F_{\Gamma \Gamma }$ using the chain rule. The superscript index (*N*) emphasizes the precedence of the local force constants. The connection between the normal bosonic operators ${\hat{A}}_{\Gamma \gamma }^{†}$ and the local bosonic operators ${\hat{a}}_{i}^{†}\left({\hat{a}}_{i}\right)$ corresponding to the internal coordinates is given by the Bogoliubov transformation

(4) $$\begin{array}{c} {\hat{A}}_{\Gamma ,\gamma }^{†}=\sqrt{\delta_{+\Gamma }}\sum_{i}m_{i,\Gamma \gamma }\;{\hat{a}}_{i}^{†}+\sqrt{\delta_{-\Gamma }}\sum_{i}m_{i,\Gamma \gamma }\;{\hat{a}}_{i}, \end{array}$$

where

(5) $$\delta_{\pm \Gamma }=\frac{1}{4}\sqrt{\frac{F_{\Gamma \Gamma }\;G_{\Gamma \Gamma }^{\left(0\right)}}{f_{qq}\;g_{qq}^{\left(0\right)}}}\left(\frac{g_{qq}^{\left(0\right)}}{G_{\Gamma \Gamma }^{\left(0\right)}}+\frac{f_{qq}}{F_{\Gamma \Gamma }}\pm 2\sqrt{\frac{f_{qq}\;g_{qq}^{\left(0\right)}}{F_{\Gamma \Gamma }\;G_{\Gamma \Gamma }^{\left(0\right)}}}\right).$$

The term $g_{qq}^{\left(0\right)}$ stands for the Wilson matrix elements in the local scheme. Given our consideration of equivalent diagonal operators, we have $f_{q_{i}q_{i}}=f_{qq};\;g_{q_{i}q_{i}}^{\left(0\right)}=g_{qq}^{\left(0\right)}\;\forall i$. The elements $\left|\right|m_{i,\Gamma \gamma }\left|\right|$ stand for the coefficients connecting the normal and local coordinates through

(6) $$Q_{\Gamma \gamma }=\sum_{i}m_{i,\Gamma \gamma }\;q_{i},$$

which are obtained by symmetry projection [34]. The relation (4) is obtained from the definition of the operators ${\hat{A}}_{\Gamma ,\gamma }^{†}$ in terms of the normal coordinates (6) and assuming bosonic operators for the local coordinates with frequencies $\omega_{i}=\sqrt{f_{q_{i}q_{i}}\;g_{q_{i}q_{i}}^{\left(0\right)}}$. Notice that, in Equation (5), there is no superscript index in the force constants $f_{qq}$. The reason for this is that the same expression will be used to estimate the force constants in both local and normal limits. The coefficients $\delta_{\pm \Gamma }$ satisfy the relation $\delta_{+\Gamma }-\delta_{-\Gamma }=1$, as a consequence of the commutator $\left[{\hat{A}}_{\Gamma^{\prime }\gamma^{\prime }},{\hat{A}}_{\Gamma \gamma }^{†}\right]=\delta_{\Gamma \Gamma^{\prime }}\delta_{\gamma \gamma^{\prime }}$. In addition, we can see that in the pure local limit $\delta_{+\Gamma }=1$ and $\delta_{-\Gamma }=0$, and consequently it is useful to introduce the average

(7) $$\delta_{-}=\frac{1}{N_{\Gamma }}\sum_{\Gamma }\delta_{-\Gamma }$$

as an LN degree, with $N_{\Gamma }$ standing for the number of irreducible representations contained in the subspace of *N* oscillators. The substitution of (4) into the Hamiltonian (2) leads to an algebraic representation of the Hamiltonian in the local scheme

(8) $$\begin{array}{c} {\hat{H}}_{L}=\sum_{i}\lambda_{0}^{\left(i\right)}\left({\hat{a}}_{i}^{†}{\hat{a}}_{i}+{\hat{a}}_{i}{\hat{a}}_{i}^{†}\right)+\sum_{i\neq j}\lambda_{1}^{\left(ij\right)}\left({\hat{a}}_{i}^{†}{\hat{a}}_{j}+{\hat{a}}_{i}{\hat{a}}_{j}^{†}\right)+\sum_{i\neq j}\lambda_{2}^{\left(ij\right)}\left({\hat{a}}_{i}^{†}{\hat{a}}_{j}^{†}+{\hat{a}}_{i}{\hat{a}}_{j}\right), \end{array}$$

with coefficients

(9a) $$\begin{array}{ccc} \lambda_{0}^{\left(i\right)} & = & \sum_{\Gamma }\frac{ћ\Omega_{\Gamma }}{2}\left(\delta_{+\Gamma }+\delta_{-\Gamma }\right)\sum_{\gamma }{\left|m_{i,\Gamma \gamma }\right|}^{2}, \end{array}$$

(9b) $$\begin{array}{ccc} \lambda_{1}^{\left(ij\right)} & = & \sum_{\Gamma }\frac{ћ\Omega_{\Gamma }}{2}\left(\delta_{+\Gamma }+\delta_{-\Gamma }\right)\sum_{\gamma }m_{i,\Gamma \gamma }m_{j,\Gamma \gamma }, \end{array}$$

(9c) $$\begin{array}{ccc} \lambda_{2}^{\left(ij\right)} & = & \sum_{\Gamma }ћ\Omega_{\Gamma }\left(\sqrt{\delta_{+\Gamma }\delta_{-\Gamma }}\right)\sum_{\gamma }m_{i,\Gamma \gamma }m_{j,\Gamma \gamma }. \end{array}$$

These expressions provide a deep insight into the problem, since it implies that, in the limit $\delta_{-}\to 0$, the coefficients $\lambda_{2}^{\left(ij\right)}$ vanish. Consequently, only when $\delta_{-}$ is negligible is the total number of local quanta $P_{L}=\sum_{i}{\hat{a}}_{i}^{†}{\hat{a}}_{i}$ conserved. We will shortly return to this point. In practice, it is more convenient to carry out the explicit substitution of (5) to express these parameters in the following form

(10a) $$\begin{array}{ccc} \lambda_{0}^{\left(i\right)} & = & \frac{ћ}{4}\sum_{\Gamma }\left(F_{\Gamma \Gamma }\sqrt{\frac{g_{qq}^{\left(0\right)}}{f_{qq}}}+G_{\Gamma \Gamma }^{\left(0\right)}\sqrt{\frac{f_{qq}}{g_{qq}^{\left(0\right)}}}\right)\sum_{\gamma }{\left|m_{i,\Gamma \gamma }\right|}^{2}, \end{array}$$

(10b) $$\begin{array}{ccc} \lambda_{1}^{\left(ij\right)} & = & \frac{ћ}{4}\sum_{\Gamma }\left(F_{\Gamma \Gamma }\sqrt{\frac{g_{qq}^{\left(0\right)}}{f_{qq}}}+G_{\Gamma \Gamma }^{\left(0\right)}\sqrt{\frac{f_{qq}}{g_{qq}^{\left(0\right)}}}\right)\sum_{\gamma }m_{i,\Gamma \gamma }m_{j,\Gamma \gamma }, \end{array}$$

(10c) $$\begin{array}{ccc} \lambda_{2}^{\left(ij\right)} & = & \frac{ћ}{4}\sum_{\Gamma }\left(F_{\Gamma \Gamma }\sqrt{\frac{g_{qq}^{\left(0\right)}}{f_{qq}}}-G_{\Gamma \Gamma }^{\left(0\right)}\sqrt{\frac{f_{qq}}{g_{qq}^{\left(0\right)}}}\right)\sum_{\gamma }m_{i,\Gamma \gamma }m_{j,\Gamma \gamma }. \end{array}$$

For equivalent oscillators the coefficients $\lambda_{0}^{\left(i\right)}$ are independent of the oscillator, and are reduced to $\lambda_{0}^{\left(i\right)}=ћ\omega_{0}/2$ with $\omega_{0}={\left(f_{qq}\;g_{qq}^{\left(0\right)}\right)}^{1/2}$ for any oscillator. The parameters $\lambda_{1}^{\left(ij\right)}$ and $\lambda_{2}^{\left(ij\right)}$ can be simplified to the form

(11) $$\lambda_{1}^{\left(ij\right)}=\frac{ћ\omega_{0}}{2}\left(x_{f}^{\left(ij\right)}+x_{g}^{\left(ij\right)}\right);\;\;\;\;\lambda_{2}^{\left(ij\right)}=\frac{ћ\omega_{0}}{2}\left(x_{f}^{\left(ij\right)}-x_{g}^{\left(ij\right)}\right),$$

with definitions

(12) $$x_{f}^{\left(ij\right)}=\frac{f_{q_{i}q_{j}}}{f_{q_{i}q_{i}}};\;\;\;\;\;\;x_{g}^{\left(ij\right)}=\frac{g_{q_{i}q_{j}}^{\left(0\right)}}{g_{q_{i}q_{i}}^{\left(0\right)}}.$$

Not all of these parameters are different. Some of them are expected to be equal, depending on the symmetry dictated by the geometrical conformation. This information is contained in the matrix $\left|\right|m_{i,\Gamma \gamma }\left|\right|$. To simplify the notation, we shall use $x_{f}^{\prime }\left(x_{g}^{\prime }\right)$ when only one type of interaction is present and $x_{f}^{\prime \prime }\left(x_{g}^{\prime \prime }\right)$ for a second type of interaction. It is important to notice that, as long as spurious modes are not present, the same Hamiltonian (8) is obtained starting from the quadratic Hamiltonian in local coordinates and momenta, which explains the subscript index *L*. The presence of redundancies imposes constraints on the local representation (8), as will be shown in Section 5.

The Hamiltonians (2) and (8) are equivalent. Their difference lies in the representation. Both Hamiltonians conserve the normal total number of quanta ${\hat{P}}_{N}=\sum_{\Gamma \gamma }{\hat{A}}_{\Gamma \gamma }^{†}{\hat{A}}_{\Gamma \gamma }$, but from the point of view of (8), ${\hat{P}}_{L}=\sum_{i}{\hat{a}}_{i}^{†}{\hat{a}}_{i}$ is not conserved. From now on, we assign the name polyad to the total number of quanta, which is justified by that fact that we shall be dealing with Hamiltonians with interactions up to second order (normal modes). In order to obtain a local polyad-conserving Hamiltonian, we have two alternatives. One possibility consists in just neglecting the interactions associated with $\lambda_{2}^{\left(ij\right)}$ in the Hamiltonian (8) to obtain

(13) $${\hat{H}}_{L}^{\left(P_{L}\right)}=\sum_{i}\lambda_{0}^{\left(i\right)}\left({\hat{a}}_{i}^{†}{\hat{a}}_{i}+{\hat{a}}_{i}{\hat{a}}_{i}^{†}\right)+\sum_{i>j}\lambda_{1}^{\left(ij\right)}\left({\hat{a}}_{i}^{†}{\hat{a}}_{j}+{\hat{a}}_{i}{\hat{a}}_{j}^{†}\right),$$

where the coefficients are kept to be identified by (10). This Hamiltonian (13) can be used to estimate the force constants

(14) $$\{f_{q_{i}q_{j}}^{\left(L\right)}\},$$

by choosing parameters to fit the fundamental energies and using the matrix representation in the local basis $|n_{1}n_{2}\ldots \rangle =\Pi_{i}⊗|n_{i}\rangle$, with $n_{i}$ being the number of local quanta for the *i*-th oscillator. It is clear that this route is feasible as long as an evident local mode behavior is present. In Table 1, analytical expressions for the force constants obtained from (3) and (11) are displayed for the systems we shall discuss.

As a second alternative to obtain a Hamiltonian with the property $[\hat{H},{\hat{P}}_{L}]=0$, we may take $\delta_{-\Gamma }=0$ in (4) and consequently also in (9). In this case, the renormalization $\delta_{+\Gamma }=1$ must be imposed to satisfy $\left[{\hat{A}}_{\Gamma^{\prime }\gamma^{\prime }},{\hat{A}}_{\Gamma \gamma }^{†}\right]=\delta_{\Gamma \Gamma^{\prime }}\delta_{\gamma \gamma^{\prime }}$ valid. In this case, the canonical transformation takes the form

(15) $$\begin{array}{c} {\hat{A}}_{\Gamma ,\gamma }^{†}=\sum_{i}m_{i,\Gamma \gamma }\;{\hat{c}}_{i}^{†}. \end{array}$$

The substitution of the operators (15) into the Hamiltonian (2) leads to the polyad-conserving Hamiltonian

(16) $${\hat{H}}_{L^{\prime }}^{\left(P_{L}\right)}=\omega_{nor}\sum_{i}\left({\hat{c}}_{i}^{†}{\hat{c}}_{i}+{\hat{c}}_{i}{\hat{c}}_{i}^{†}\right)+\sum_{i\neq j}\lambda_{nor}^{\left(ij\right)}\left({\hat{c}}_{i}^{†}{\hat{c}}_{j}+{\hat{c}}_{i}{\hat{c}}_{j}^{†}\right),$$

with coefficients

(17a) $$\begin{array}{ccc} \omega_{nor} & = & \sum_{\Gamma }\frac{ћ\Omega_{\Gamma }}{2}\sum_{\gamma }{\left|m_{i,\Gamma \gamma }\right|}^{2}, \end{array}$$

(17b) $$\begin{array}{ccc} \lambda_{nor}^{\left(ij\right)} & = & \sum_{\Gamma }\frac{ћ\Omega_{\Gamma }}{2}\sum_{\gamma }m_{i,\Gamma \gamma }m_{j,\Gamma \gamma }, \end{array}$$

The Hamiltonians (16) and (8) look similar when $\lambda_{2}^{\left(ij\right)}=0$, but they are not the same. Two features make them different: (i) the bosonic operators ${\hat{c}}_{i}^{†}\left({\hat{c}}_{i}\right)$ are not strictly local, which explains the subscript index $L^{\prime }$; and (ii) the relation between the spectroscopic parameters and the force constants is different, a fact that suggests an additional LN criterion, as we next discuss.

The sets of parameters $\left\{\omega_{nor},\lambda_{nor}^{\left(ij\right)}\right\}$ in (16) and $\left\{\lambda_{0}^{\left(i\right)},\lambda_{1}^{\left(ij\right)}\right\}$ in (13) are functions of both the force and the structure constants, although with different functional form. To establish the connection between both sets, it is convenient to recall the definitions (12), since they are expected to be small, actually vanishing at the pure local limit. The set $\left\{\lambda_{0}^{\left(i\right)},\lambda_{1}^{\left(ij\right)}\right\}$ is expected to be recovered from $\left\{\omega_{nor},\lambda_{nor}^{\left(ij\right)}\right\}$ near the local limit. The latter set can be considered a function of the variables (12), which can be put together in vector form $x=\{x_{f}^{\left(ij\right)},\ldots ,x_{g}^{\left(ij\right)},\ldots \}$. The connection between the parameters is given by the Taylor series expansion

(18a) $$\begin{array}{ccc} \omega_{nor} & = & \frac{2\lambda_{0}^{\left(i\right)}}{ћ}\left[1+\frac{1}{2!}xH^{\omega }\left(0\right)\tilde{x}+{\left||x|\right|}^{2}E_{2}^{\omega }\left(0,x\right)\right], \end{array}$$

(18b) $$\begin{array}{ccc} \lambda_{nor}^{\left(ij\right)} & = & \lambda_{1}^{\left(ij\right)}+\omega_{loc}\left[\frac{1}{2!}xH^{\lambda^{\left(ij\right)}}\left(0\right)\tilde{x}+{\left||x|\right|}^{2}E_{2}^{\lambda^{\left(ij\right)}}\left(0,x\right)\right], \end{array}$$

where H(0) is the Hessian matrix evaluated at x=0 and ${\left||x|\right|}^{2}E_{2}\left(0,x\right)$ denotes the error involved up to second order. It is clear that the second-order terms measure the deviation of the parameters from the local mode description, but they also indicate the degree of local polyad conservation. We thus propose the set

(19) $$\gamma^{\left(\omega \right)}=|\frac{1}{2!}xH^{\omega }\left(0\right)\tilde{x}|;\;\;\;\gamma^{\left(ij\right)}=|\frac{1}{2!}xH^{\lambda^{\left(ij\right)}}\left(0\right)\tilde{x}|,$$

as new parameters to provide an LN degree. In addition, the spectroscopic parameters (11) from the local scheme and the parameters involved in (16) provide different force constants, both connected through (18). This fact suggests the introduction of the following parameters to estimate the LN degree

(20) $$\epsilon_{1}=1-\frac{f_{qq}^{\left(L\right)}}{f_{qq}^{\left(N\right)}};\;\;\epsilon^{\left(ij\right)}=1-\frac{f_{q_{i}q_{j}}^{\left(L\right)}}{f_{q_{i}q_{j}}^{\left(N\right)}},$$

since $\lim_{x\to 0}\epsilon_{1}=\epsilon^{\left(ij\right)}=0$.

On the other hand, since the energy splitting of a set of degenerate equivalent oscillators is expected to be proportional to the their interaction strength, the following LN parameter may also be proposed [30,31]

(21) $$\zeta =|\frac{2}{\pi }\arctan\left(\frac{E_{d}-E_{u}}{(E_{d}+E_{u})/2}\right)|$$

where $E_{d}$ and $E_{u}$ correspond to the lowest and highest energy of the multiplet.

Summarizing, we have identified four parameters, namely $\delta_{-\Gamma },\gamma ,\left\{\epsilon_{i},\epsilon^{\left(ij\right)}\right\}$, and ζ, which vanish in the local limit, and consequently provide a way to measure the locality(normality) of a molecule. It is convenient to emphasize that these criteria do not assume a model of interacting Morse oscillators, but only harmonic oscillators. They arise from bosonic operators and are determined by the fundamental energies. We now describe their characteristics:

$\delta_{-\Gamma }$: This parameter measures the suitability of applying the polyad-conserving canonical transformation (15) for each symmetry (normal mode). For convenience, we also introduced the average (7), which takes into account the contribution of all normal modes. It is worth stressing that this parameter $\delta_{-}$ can be calculated for any molecular system, and from its definition (5) it measures the degree of locality/normality from the point of view of the normal mode scheme. In contrast, the polyad-conserving transformation (15) has been assumed in every study of the local-to-normal mode transition involving stretching degrees of freedom of molecules with a clear local mode behavior. Indeed, this assumption leads to the *x*-*K* relations [18,35,36,37,38,39,40,41,42], which stop being valid when the molecules move to a normal mode behavior.

$\gamma^{\prime }s$: These parameters correspond to the Hessians in (18) and they provide the quadratic approximation for the sets $\left\{\omega_{nor},\lambda_{nor}^{\left(ij\right)}\right\}$ starting from $\left\{\lambda_{0}^{\left(i\right)},\lambda_{1}^{\left(ij\right)}\right\}$ when the local parameters become small. These parameters establish LN criteria from the point of view of the local mode scheme and are expected to be correlated with $\delta_{-}$, in some cases in a perfect linear trend when the higher order terms in the expansion (18) are neglected compared with the quadratic terms, as we shall discuss.

$\epsilon^{\prime }s$: This set of parameters are introduced to see the impact of the LN degree on the estimation of the force constants and takes into account both the normal and local mode schemes, since both estimations (3) and (14) for the force constants are involved. However, in order to obtain reliable results, the force constants should not be too small, since the errors may hide the criterion.

ζ: This parameter takes into account the correlation between the strength of the interaction and the LN degree, and it is a natural parameter based on the correlation between the splitting of the fundamentals and the strength of the interaction. However, we shall prove that the parameter ζ, although intuitive, is not appropriate for establishing a LN degree.

Here, we have assumed that the development of this approach lies in the formulation of the model in terms of internal coordinates. This route can be quite elaborate from a theoretical point of view because of the calculation of the Wilson matrix and the identification of redundancies. However, since these parameters involve the concept of normal modes, the calculations are quite fast.

A comment regarding the correlation between the polyad breaking and the LN degree deserves special attention. The canonical transformation (15) conserves the local polyad, but even when the local polyad is not conserved, the Hamiltonian satisfies $[{\hat{P}}_{N},H_{L}]=0$. Substitution of (4) into the definition of a normal polyad leads to the relation

(22) $$\begin{array}{ccc} {\hat{P}}_{N} & = & \zeta_{0}+\sum_{i}\beta_{0}^{\left(i\right)}\;\left({\hat{a}}_{i}^{†}{\hat{a}}_{i}\right)+\sum_{i\neq j}\beta_{1}^{\left(ij\right)}\;\left({\hat{a}}_{i}^{†}{\hat{a}}_{j}+{\hat{a}}_{i}{\hat{a}}^{†}{+}_{j}\right)\\ & + & \sum_{i}\beta_{2}^{\left(i\right)}\;\left({\hat{a}}_{i}^{†2}+{\hat{a}}_{i}^{2}\right)+\sum_{i\neq j}\beta_{3}^{\left(ij\right)}\left({\hat{a}}_{i}^{†}{\hat{a}}_{j}^{†}+{\hat{a}}_{i}{\hat{a}}_{j}\right), \end{array}$$

with coefficients given by

(23a) $$\begin{array}{ccc} \zeta_{0} & = & N_{\Gamma }\sum_{\Gamma }\delta_{-\Gamma }=N_{\Gamma }^{2}\delta_{-}, \end{array}$$

(23b) $$\begin{array}{ccc} \beta_{0}^{\left(i\right)} & = & \sum_{\Gamma \gamma }\left(\delta_{+\Gamma }+\delta_{-\Gamma }\right){\left|m_{i,\Gamma \gamma }\right|}^{2}, \end{array}$$

(23c) $$\begin{array}{ccc} \beta_{1}^{\left(ij\right)} & = & \sum_{\Gamma \gamma }\left(\delta_{+\Gamma }+\delta_{-\Gamma }\right)\;m_{i,\Gamma \gamma }m_{j,\Gamma \gamma }, \end{array}$$

(23d) $$\begin{array}{ccc} \beta_{2}^{\left(i\right)} & = & \sum_{\Gamma \gamma }\sqrt{\delta_{+\Gamma }\delta_{-\Gamma }}{\left|m_{i,\Gamma \gamma }\right|}^{2}, \end{array}$$

(23e) $$\begin{array}{ccc} \beta_{3}^{\left(ij\right)} & = & \sum_{\Gamma \gamma }\sqrt{\delta_{+\Gamma }\delta_{-\Gamma }}\;m_{i,\Gamma \gamma }\;m_{j,\Gamma \gamma }. \end{array}$$

The transformation (15) assumes $\delta_{-\Gamma }=0$ and $\delta_{+\Gamma }=1$, leading to the values $\zeta_{0}=\beta_{1}^{\left(ij\right)}=\beta_{2}^{\left(ij\right)}=\beta_{3}^{\left(ij\right)}=0$ and $\beta_{0}^{\left(ij\right)}=1$, with ${\hat{P}}_{N}={\hat{P}}_{L}$. The explicit behavior of these parameters will be studied later on in the context of two equivalent oscillators. At the moment, we just reinforce the argument that local polyad breaking is strongly correlated with the LN degree.

In the next sections, we present an analysis for different numbers of oscillators. The aim is to show that the correlation between different parameters unveils physical properties. Due to its importance, the case of two oscillators will be studied in detail, including the case of non-equivalent oscillators. The latter illustrates the way our approach is modified when the normal mode coordinates do not coincide with symmetry-adapted coordinates.

## 3. LN Degree in Triatomic Molecules

In this section, we revisit the stretching degrees of freedom of triatomic molecules. Since this system has already been discussed in part [30,31], we present only its salient features, although in modified form in accordance with the general framework presented in the previous section. First, we consider the case of equivalent oscillators.

### 3.1. Equivalent Oscillators

For two equivalent oscillators, the irreducible representations are two, which we name as Γ=g,u. The expressions for the force constants (3) are [30,31]

(24a) $$\begin{array}{ccc} F_{gg} & = & f_{rr}+f_{rr^{\prime }};\;G_{gg}^{\left(0\right)}=g_{rr}^{\left(0\right)}+g_{rr^{\prime }}^{\left(0\right)}; \end{array}$$

(24b) $$\begin{array}{ccc} F_{uu} & = & f_{rr}-f_{rr^{\prime }},\;G_{uu}^{\left(0\right)}=g_{rr}^{\left(0\right)}-g_{rr^{\prime }}^{\left(0\right)}, \end{array}$$

from which we obtain the force constants $f_{q_{i}q_{j}}^{\left(N\right)}$ provided by Table 1, with $\mu_{2}=u$. The parameters (5) can be simplified to

(25) $$\delta_{\pm \Gamma }=\frac{1}{4}\frac{{\left(r_{\Gamma }\pm 1\right)}^{2}}{r_{\Gamma }};\;\;\;\;\;\;r_{\Gamma }=\sqrt{\frac{1+{\left(-\right)}^{\sigma_{\Gamma }}x_{f}^{\prime }}{1+{\left(-\right)}^{\sigma_{\Gamma }}x_{g}^{\prime }}},$$

where $\sigma_{g}=0$ and $\sigma_{u}=1$. The matrix $\left|\right|m_{i,\Gamma \gamma }\left|\right|$ corresponding to (6) takes the form

(26) $$\left|\right|m_{i,\Gamma \gamma }\left|\right|=\frac{1}{\sqrt{2}}\left(\begin{array}{cc} 1 & 1\\ 1 & -1 \end{array}\right).$$

The substitution of (4) into (2) leads to an algebraic representation in terms of local bosonic operators ${\hat{a}}_{i}^{†}\left({\hat{a}}_{i}\right)$:

(27) $$\begin{array}{c} {\hat{H}}_{L}=\lambda_{0}\sum_{i=1}^{2}\left({\hat{a}}_{i}^{†}{\hat{a}}_{i}+{\hat{a}}_{i}{\hat{a}}_{i}^{†}\right)+\lambda_{1}^{\left(1\right)}\left({\hat{a}}_{1}^{†}{\hat{a}}_{2}+{\hat{a}}_{1}{\hat{a}}_{2}^{†}\right)+\lambda_{2}^{\left(1\right)}\left({\hat{a}}_{1}^{†}{\hat{a}}_{2}^{†}+{\hat{a}}_{1}{\hat{a}}_{2}\right), \end{array}$$

with coefficients given by (11). Here, the superscript index (1) in λ’s indicates that only one type of interaction is present. The basis for constructing the matrix representation of the Hamiltonian (27) is given by the direct product $|n_{1}n_{2}\rangle \;=\;|n_{1}\rangle \;⊗\;|n_{2}\rangle$. When the interaction strength $\lambda_{2}^{\left(1\right)}$ is large, the whole space of states $|n_{1}n_{2}\rangle$ is mixed, leading to a time-consuming diagonalization. In contrast, when $\lambda_{2}^{\left(1\right)}$ is negligible, the Hamiltonian commutes with the operator ${\hat{P}}_{L}$. Hence, if we make the approximation $\lambda_{2}^{\left(1\right)}\to 0$ and fit the $\lambda_{0}$ and $\lambda_{1}^{\left(1\right)}$ to reproduce the fundamentals, we can estimate the force constants (14) from (11). The analytical expressions are given in Table 1.

A more fruitful viewpoint for obtaining a local polyad-conserving Hamiltonian is provided by realizing that $\lambda_{2}^{\left(1\right)}$ also vanishes when the contribution of annihilation operators in (4) is null. To follow this route, we apply the canonical transformation (15), which when substituted into (2), leads to the Hamiltonian

(28) $${\hat{H}}_{L^{\prime }}^{\left(P_{L}\right)}=\omega_{nor}\sum_{i=1}^{2}\left({\hat{c}}_{i}^{†}{\hat{c}}_{i}+{\hat{c}}_{i}{\hat{c}}_{i}^{†}\right)+\lambda_{nor}^{\left(1\right)}\left({\hat{c}}_{1}^{†}{\hat{c}}_{2}+{\hat{c}}_{1}{\hat{c}}_{2}^{†}\right),$$

where

(29a) $$\begin{array}{ccc} \omega_{nor}= & \frac{ћ\omega_{0}}{2} & \left(\frac{1}{2}\sqrt{\left(1+x_{f}^{\prime }\right)\left(1+x_{g}^{\prime }\right)}+\frac{1}{2}\sqrt{\left(1-x_{f}^{\prime }\right)\left(1-x_{g}^{\prime }\right)}\right) \end{array}$$

(29b) $$\begin{array}{ccc} \lambda_{nor}^{\left(1\right)}= & \frac{ћ\omega_{0}}{2} & \left(\sqrt{\left(1+x_{f}^{\prime }\right)\left(1+x_{g}^{\prime }\right)}-\sqrt{\left(1-x_{f}^{\prime }\right)\left(1-x_{g}^{\prime }\right)}\right). \end{array}$$

The relation between these coefficients and (29) is obtained through the Equation (18), which takes the form [30,31]

(30) $$\begin{array}{cc} \omega_{nor} & =\frac{ћ\omega_{0}}{2}\left(1-\frac{1}{8}{\left(x_{g}^{\prime }-x_{f}^{\prime }\right)}^{2}+O\left(x^{3}\right)\right)\;, \end{array}$$

(31) $$\begin{array}{cc} \lambda_{nor}^{\left(1\right)} & =\;\frac{ћ\omega_{0}}{2}\left(x_{g}^{\prime }+x_{f}^{\prime }+O\left(x^{3}\right)\right)\;. \end{array}$$

with the identification

(32) $$\gamma^{\left(\omega \right)}=\frac{1}{8}{\left(x_{f}^{\prime }-x_{g}^{\prime }\right)}^{2}.$$

This parameter is related to $\lambda_{2}^{\left(1\right)}$ appearing in the Hamiltonian (27) by $\gamma^{\left(\omega \right)}=\frac{1}{2}{\left(\frac{\lambda_{2}^{\left(1\right)}}{ћ\omega_{0}}\right)}^{2}$, which is consistent with the fact that both of them are associated with local polyad breaking. From the expressions for the force constants obtained from Table 1, we can calculate the parameters $\epsilon_{i},i=1,2$ defined in (20). Finally, we should recall that we have the additional parameter ζ defined in (21), which was used in Refs. [30,31] for the case of two oscillators.

**Figure 1:**
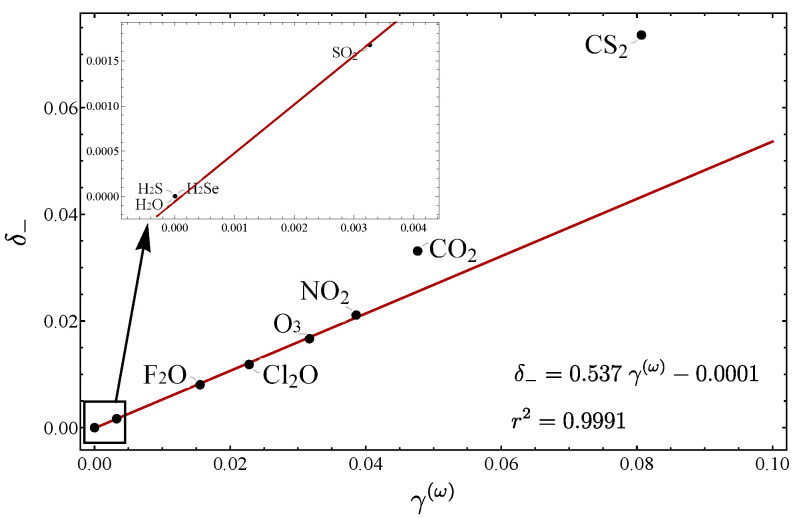
Plot of the parameters $\delta_{-}$ vs. γ for the series of molecules included in Table 3.

We now proceed to show the relation between these parameters for different molecules. We start by considering the series of symmetric triatomic molecules analyzed in Refs. [30,31]. In Table 2, the fundamentals as well as the force constants are displayed, while in Table 3 all the parameters suitable for measuring the LN degree are listed. In order to appreciate the behavior of the parameters, it is convenient to display the results in graphical form. In Refs. [30,31], the plot ζ vs. γ was presented. Although a general local-to-normal trend was identified, a clear correlation between the parameters was not manifested. This fact suggests that the splitting of the interacting oscillators is not necessarily a quantitative criterion for assigning an LN degree. In Figure 1, a plot of $\delta_{-}$ vs. $\gamma^{\left(\omega \right)}$ for all the molecules included in Table 3 is depicted. Now, we can see a linear trend for the molecules near the local limit. The molecules with normal mode behavior are shifted to the upper part of the line. The reason for this is that the parameter $\gamma^{\left(\omega \right)}$ only takes into account the first term of the expansion (30). A discussion of this behavior will be provided in Section 3.3. The importance of the correlation between $\delta_{-}$ and $\gamma^{\left(\omega \right)}$ is two fold: first both parameters represent a consistent LN criterion and second this correlation allows unveiling molecular properties, as we shall see later on.

**Figure 2:**
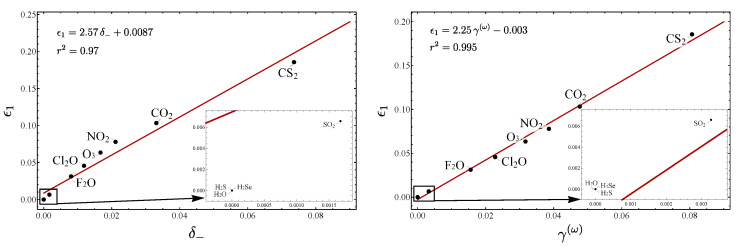
Plots of $\epsilon_{1}$ vs. $\delta_{-}$ and $\epsilon_{1}$ vs. $\gamma^{\left(\omega \right)}$, for the same series of molecules considered in Figure 1.

We now turn our attention to the correlation between the parameters $\epsilon_{1}$ vs. $\delta_{-}$ and $\epsilon_{1}$ vs. $\gamma^{\left(\omega \right)}$ displayed in Figure 2. A clear linear trend is obtained, which confirms the validity of the parameter $\epsilon_{1}$ for measuring the LN degree.

Regarding the correlation of $\epsilon_{2}$ vs. $\delta_{-}$, a somewhat unclear behavior is obtained for the molecules with strong local behavior. This is explained as, in the region close to locality, a weak interaction between oscillators is present, leading to small values of $f_{rr^{\prime }}$. This fact makes it difficult to assign an LN degree; the error is of the same order of magnitude as the parameters themselves.

### 3.2. Non-Equivalent Bonds

In the previous analysis, we considered two equivalent oscillators, a case where the principal isotopologues are embraced. However, we can incorporate the full variety of triatomic molecules through a symmetry reduction. In this case, it is more convenient to start with an algebraic representation of the Hamiltonian in terms of local operators.

(33) $$\begin{array}{c} {\hat{H}}_{L}=\frac{ћ\omega_{1}}{2}\left\{\left({\hat{a}}_{1}^{†}{\hat{a}}_{1}+{\hat{a}}_{1}{\hat{a}}_{1}^{†}\right)+\sqrt{\alpha \beta }\left({\hat{a}}_{2}^{†}{\hat{a}}_{2}+{\hat{a}}_{2}{\hat{a}}_{2}^{†}\right)\lambda_{1}^{\left(1\right)}\left({\hat{a}}_{1}^{†}{\hat{a}}_{2}+{\hat{a}}_{1}{\hat{a}}_{2}^{†}\right)+\lambda_{2}^{\left(1\right)}\left({\hat{a}}_{1}^{†}{\hat{a}}_{2}^{†}+{\hat{a}}_{1}{\hat{a}}_{2}\right)\right\}, \end{array}$$

with the new definitions

(34) $$\omega_{1}=\sqrt{f_{11}g_{11}^{\left(0\right)}}\;;\;\lambda_{1}^{\left(1\right)}=\sqrt{\alpha \beta }\left(x_{f}^{\prime }+x_{g}^{\prime }\right)\;;\;\lambda_{2}^{\left(1\right)}=\sqrt{\alpha \beta }\left(x_{f}^{\prime }-x_{g}^{\prime }\right)$$

and

(35) $$x_{f}^{\prime }=\frac{f_{12}}{\sqrt{f_{11}f_{22}}}\;;\;x_{g}^{\prime }=\frac{g_{12}^{\left(0\right)}}{\sqrt{g_{11}^{\left(0\right)}g_{22}^{\left(0\right)}}}\;;\;\alpha^{2}=\frac{g_{22}^{\left(0\right)}}{g_{11}^{\left(0\right)}}\;;\;\beta^{2}=\frac{f_{22}}{f_{11}}.$$

The algebraic Hamiltonian (33) is obtained from the quadratic Hamiltonian for two interacting oscillators in configuration space. For the general case like FCN, α≠1 and β≠1. Assuming the Born–Oppenheimer approximation, for asymmetric isotopologues of type ${}^{x_{1}}B^{y}A^{x_{2}}B$, we have β=1, while for symmetric molecules, it is clear that α=β=1; the case previously analyzed.

We proceed to obtain the normal representation of the Hamiltonian (33). To consider this scenario, we invoke the GF formalism to obtain the normal modes. The normal modes are defined in terms of the internal coordinates using the transformation $L^{-1}G_{0}FL=\Lambda$, where the matrices $G_{0}$ and F are chosen in terms of the symmetry-adapted coordinates $S=\{S_{g}=\left(q_{1}+q_{2}\right)/\sqrt{2},S_{u}=\left(q_{1}-q_{2}\right)/\sqrt{2}\}$ [33]. Here, the condition $L^{†}G_{0}^{-1}L=1$ provides the normalization of L.

In the normal mode scheme, the algebraic Hamiltonian equivalent to (2) takes the general form

(36) $$\begin{array}{ccc} {\hat{H}}_{N} & = & \sum_{\iota }\frac{ћ\Omega_{\iota (\Gamma )}}{2}\sum_{\gamma }\left({\hat{A}}_{\iota \gamma }^{†}{\hat{A}}_{\iota \gamma }+{\hat{A}}_{\iota \gamma }{\hat{A}}_{\iota \gamma }^{†}\right), \end{array}$$

where we have taken into account that the ι-th normal mode carries the Γ-th irreducible representation with components γ. For the particular case of two non-equivalent interacting oscillators, the Hamiltonian (36) simplifies to

(37) $$\begin{array}{ccc} {\hat{H}}_{N} & = & \sum_{\iota }\frac{ћ\Omega_{\iota (\Gamma )}}{2}\left({\hat{A}}_{\iota }^{†}{\hat{A}}_{\iota }+{\hat{A}}_{\iota }{\hat{A}}_{\iota }^{†}\right), \end{array}$$

with Γ identified with the totally symmetric irreducible representation. It was found that the connection between the bosonic normal operators ${\hat{A}}_{\iota }^{†}\left({\hat{A}}_{\iota }\right)$ associated with the ι-th normal mode and the local operators ${\hat{a}}_{j}^{†}\left({\hat{a}}_{j}\right)$ is the following [33]

(38) $${\hat{A}}_{\iota }^{†}=\sum_{j=1}^{2}\left(c_{+}^{\left(\iota ,j\right)}{\hat{a}}_{j}^{†}+c_{-}^{\left(\iota ,j\right)}{\hat{a}}_{j}\right),$$

with coefficients

(39) $$\begin{array}{c} c_{\pm }^{\left(\iota ,j\right)}=\frac{1}{2\sqrt{2}}\sum_{\sigma }{\left(-1\right)}^{\left(\sigma +1\right)\left(1-\delta_{j1}\right)}\beta_{\iota }\sqrt{\frac{ћ}{\omega_{j}\mu_{j}}}\left[{\left(L^{-1}\right)}_{\iota \sigma }\pm {\left(\tilde{L}\right)}_{\iota \sigma }\frac{\omega_{j}\mu_{j}}{ћ\beta_{\iota }^{2}}\right]. \end{array}$$

where $\beta_{\iota }^{2}=\frac{1}{ћ}{\left(\lambda_{\iota }\right)}^{1/2}$, with $\lambda_{\iota }=\Lambda_{\iota \iota }$ given by

(40) $$\lambda_{\pm }=\frac{g_{11}f_{11}}{2}\left(1+\eta^{2}+2\eta x_{f}^{\prime }x_{g}^{\prime }\pm \sqrt{1+\eta^{2}\left(\eta^{2}-2\right)+4\eta \left[\eta \left(x_{f}^{\prime 2}+x_{g}^{\prime 2}\right)+\left(1+\eta^{2}\right)x_{f}^{\prime }x_{g}^{\prime }\right]}\right),$$

with η=αβ. In Equation (39), σ denotes a sum over the symmetry-adapted coordinates σ={1→g,2→u}. The matrix L is defined through S=LQ:

(41) $$Q_{\iota }=\sum_{\alpha }{\left(L^{-1}\right)}_{\iota \alpha }S_{\alpha };\;\;\;\alpha =1,2.$$

If we now into account that $\mu_{j}=1/g_{q_{j}q_{j}}^{\left(0\right)}$ and $\omega_{j}=\sqrt{f_{q_{j}q_{j}}g_{q_{j}q_{j}}^{\left(0\right)}}$, Equation (38) allows the parameter $\delta_{-}$ to be defined

(42) $$\delta_{-}=\frac{1}{2}\sum_{\iota =1}^{2}\left[\sum_{j=1}^{2}{\left|c_{-}^{\left(\iota ,j\right)}\right|}^{2}\right],$$

which measures the LN degree and also the feasibility of establishing a polyad preserving canonical transformation from (38). Here, we have to stress that, because of the non-equivalence between the oscillators, the coefficients $c_{\pm }^{\left(\iota j\right)}$ cannot be factorized as happened in (4).

In order to identify the parameters (19), we recall the connection between (32) and $\lambda_{2}^{\left(1\right)}$ in (27). Hence, from $\lambda_{2}^{\left(12\right)}\to \lambda_{2}^{\left(1\right)}$, we define

(43) $$\gamma^{\left(\omega \right)}=\frac{1}{8}{\left(x_{f}^{\prime }-x_{g}^{\prime }\right)}^{2},$$

as the parameter connected with the Hessian in (18), albeit with the new definitions (35).

As we know, the substitution of (38) into the Hamiltonian (2) leads to the algebraic Hamiltonian (33) given in the local representation. But now, in order to obtain the equivalent expression to (28), we need to consider the polyad-conserving canonical transformations

(44a) $$\begin{array}{ccc} {\hat{A}}_{1}^{†} & = & \alpha {\hat{c}}_{1}^{†}+\sqrt{1-\alpha^{2}}{\hat{c}}_{2}^{†} \end{array}$$

(44b) $$\begin{array}{ccc} {\hat{A}}_{2}^{†} & = & \sqrt{1-\alpha^{2}}{\hat{c}}_{1}^{†}-\alpha {\hat{c}}_{2}^{†} \end{array}$$

to ensure the commutation relations $\left[{\hat{A}}_{\iota },{\hat{A}}_{\iota^{\prime }}^{†}\right]=\delta_{\iota \iota^{\prime }},$ where α is chosen to be $\alpha =c_{+}^{\left(1,1\right)}$. Substitution of (44) into (2) gives rise to the Hamiltonian

(45) $$\begin{array}{c} {\hat{H}}_{L^{\prime }}^{\left(P_{L}\right)}=\omega_{nor}^{\left(1\right)}\left({\hat{c}}_{1}^{†}{\hat{c}}_{1}+{\hat{c}}_{1}{\hat{c}}_{1}^{†}\right)+\omega_{nor}^{\left(2\right)}\left({\hat{c}}_{2}^{†}{\hat{c}}_{2}+{\hat{c}}_{2}{\hat{c}}_{2}^{†}\right)+\lambda_{nor}^{\left(12\right)}\left({\hat{c}}_{1}^{†}{\hat{c}}_{2}+{\hat{c}}_{1}{\hat{c}}_{2}^{†}\right), \end{array}$$

where

(46a) $$\begin{array}{ccc} \omega_{nor}^{\left(1\right)} & = & \frac{ћ}{2}\left(\Omega_{1}\;\alpha^{2}+\Omega_{2}\;\left(1-\alpha^{2}\right)\right) \end{array}$$

(46b) $$\begin{array}{ccc} \omega_{nor}^{\left(2\right)} & = & \frac{ћ}{2}\left(\Omega_{1}\;\left(1-\alpha^{2}\right)+\Omega_{2}\;\alpha^{2}\right) \end{array}$$

(46c) $$\begin{array}{ccc} \lambda_{nor}^{\left(12\right)} & = & ћ\alpha \sqrt{1-\alpha^{2}}\left(\Omega_{1}-\Omega_{2}\right). \end{array}$$

Both the Hamiltonians (33) and (45) involve three force constants $\left\{f_{11},f_{22},f_{12}\right\}$. This is indeed the case in a general situation like the FCN molecule, where the two fundamentals are not enough to determine them. In such situations, fundamentals for an isotopologue are needed to estimate the three force constants. For symmetric isotopologues $f_{11}=f_{22}$, and just the two fundamentals need to be determined.

The transformation (44) acquires a preponderant importance in describing molecules with clear normal mode behavior, like CO${}_{2}$, N${}_{2}$O, OCS, or FCN, for instance. In molecules with local mode behavior, the usual approach consists in describing the stretching internal coordinates in terms of interacting Morse oscillators, with the bends in terms of normal coordinates. The splitting of the stretching fundamental energies is small and consequently a local polyad-conserving Hamiltonian may be a good approximation. In contrast, for the bending degrees of freedom , this is not possible because the polyad is only well defined in the normal scheme. The same situation is present for the stretching degrees of freedom in molecules with strong normal behavior. In order to embrace both behaviors, we propose to apply the transformation (44) to a normal polyad Hamiltonian to obtain a local representation. The obtained Hamiltonian is later anharmonized by mapping the bosonic operators ${\hat{c}}_{i}^{†}\left({\hat{c}}_{i}\right)$ for stretches to SU(2) operators ${\hat{b}}_{i}^{†}\left({\hat{b}}_{i}\right)$ associated with Morse ladder operators. This approach allows us to take advantage of the Morse properties in systems where only the normal polyad is defined. This procedure has been tested on the whole series of isotopologues of carbon dioxide [33,53] and the FCN molecule [54].

### 3.3. Parameterization Local–Normal Mode Transition

**Figure 3:**
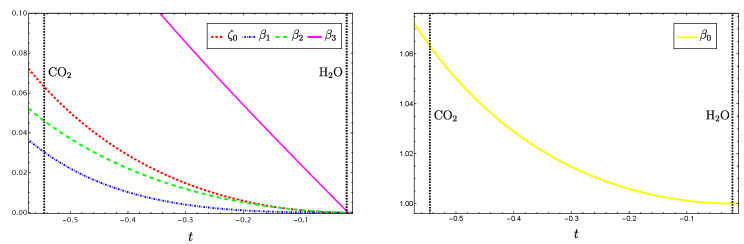
Coefficients involved in the relation (22) connecting the normal and local polyads.

Because of the close relation between the contribution of the annihilation operators in (38) and local polyad breaking ${\hat{P}}_{L}$, it is convenient to recall the parameterization from H_2_O to CO${}_{2}$ presented in Refs. [30,31], but now taking care of the linear path appearing in the diagram $\delta_{-}$ vs. γ. Both parameters depend on $x_{g}^{\prime }$ and $x_{f}^{\prime }$. Hence, taking $t\equiv x_{g}^{\prime }$, we have for $x_{f}^{\prime }\left(t\right)$

(47a) $$\begin{array}{ccc} x_{f}^{\prime }\left(t\right) & = & m_{f}\;\left(t-x_{g}^{\prime (N)}\right)+x_{f}^{\prime (N)};\;\;m_{f}=\frac{x_{f}^{\prime (N)}-x_{f}^{\prime (L)}}{x_{g}^{\prime (N)}-x_{g}^{\prime (L)}}, \end{array}$$

(47b) $$\begin{array}{ccc} \omega (t) & = & m_{\omega }\left(t-x_{g}^{\prime (N)}\right)+\omega^{\left(N\right)};\;\;m_{\omega }=\frac{\omega^{\left(N\right)}-\omega^{\left(L\right)}}{x_{g}^{\prime (N)}-x_{g}^{\prime (L)}}, \end{array}$$

with $t\in [x_{g}^{\prime (L)},x_{g}^{\prime (N)}]$, while $\left(x_{g}^{\prime (L)},x_{f}^{\prime (L)}\right)$ and $\left(x_{g}^{\prime (N)},x_{f}^{\prime (N)}\right)$ correspond to the values of the parameters for the molecules H${}_{2}$O and CO${}_{2}$, respectively. This parametrization can be used to study Equation (22) connecting the normal and local polyads. From Figure 3, we can see that, at the local limit, all parameters vanish, with the exception of $\beta_{0}$ going to unity and leading to ${\hat{P}}_{N}={\hat{P}}_{L}$, as expected. We found that these parameters are basically correlated to the $\delta_{-}$ parameter and consequently they do not contribute to additional LN criteria.

**Figure 4:**
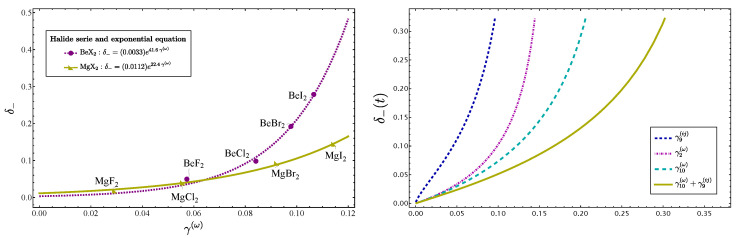
At the left, the plot $\delta_{-}$ vs. $\gamma^{\left(\omega \right)}$ is displayed, including the series of molecules BeX${}_{2}$ and MgX${}_{2}$, which manifest a strong normal mode behavior. On the right, the equivalent plot using the parameterization (47), but adding to $\gamma^{\left(\omega \right)}$ additional contributions up to *n*-th order indicated with $\gamma_{n}^{\left(\omega \right)}$. The curve labeled $\gamma_{10}^{\left(\omega \right)}+\gamma_{9}^{\left(ij\right)}$ means that both gammas were summed up the indicated order.

Up to now, we have considered the correlation of $\delta_{-}$ vs. $\gamma^{\left(\omega \right)}$ for molecules near a local mode behavior. Figure 1 suggests that the linear trend is a consequence of the local mode behavior, since the location of CO${}_{2}$ and CS${}_{2}$ lies outside of the linear correlation. This situation is clearly manifested in Figure 4, left side, where the series of molecules BeX${}_{2}$ and MgX${}_{2}$ are analyzed. We can see that by including molecules with strong normal mode behavior the function changes from linear to exponential form, making it clear that the linear correlation is just an approximation. To explain this behavior, we notice that from the point of view of the series (18), $\gamma^{\left(\omega \right)}$ represents the first correction to the linear approximation. When higher-order terms are incorporated, the linear trend tends to be recovered, as seen in the same Figure 4 on the right-hand side. This behavior also explains the location of the molecules CO${}_{2}$ and CS${}_{2}$ above the line in Figure 1. On the other hand, the parameter $\delta_{-}$ is unique, while there are several $\gamma^{\prime }s$. This fact suggests that putting together the information of all the $\gamma^{\prime }s$, a closer approximation to $\delta_{-}$ is obtained. This guess is confirmed by the plot of $\delta_{-}$ vs. $\gamma_{10}^{\left(\omega \right)}+\gamma_{9}^{\left(ij\right)}$ depicted on the right of Figure 4. The subscript index in the γ’s means the upper order is taken into account. In our analysis, the addition of higher-order terms to $\gamma^{\left(\omega \right)}$ is not necessary to define a different parameter, because of the clear exponential form, which ensures a consistent behavior, and consequently both $\delta_{-}$ and $\gamma^{\left(\omega \right)}$ provide LN parameters, even in extreme conditions of normality.

### 3.4. Isotopologues

The analysis of the series of isotopologues deserves special attention, because the plots $\delta_{-}$ vs. $\gamma^{\left(\omega \right)}$ provide a perfect linear correlation, which is expected from Figure 4, since they represent a short segment of the exponential function. In addition, since each series of isotopologues is characterized using the same force constants, the slopes are expected to be correlated to $x_{f}^{\prime }$.

**Figure 5:**
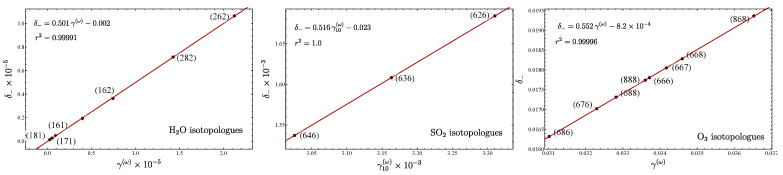
Plots of $\delta_{-}$ vs. $\gamma^{\left(\omega \right)}$ for the series of isotopologues of H${}_{2}$O, SO${}_{2}$, and O${}_{3}$. A clear linear trend was obtained in all systems. The fundamentals needed to obtain the parameters were obtained from the references indicated: water [55,56,57], sulfur dioxide [58], and ozone [49]. The force constants were obtained from the principal isotopologues.

**Figure 6:**
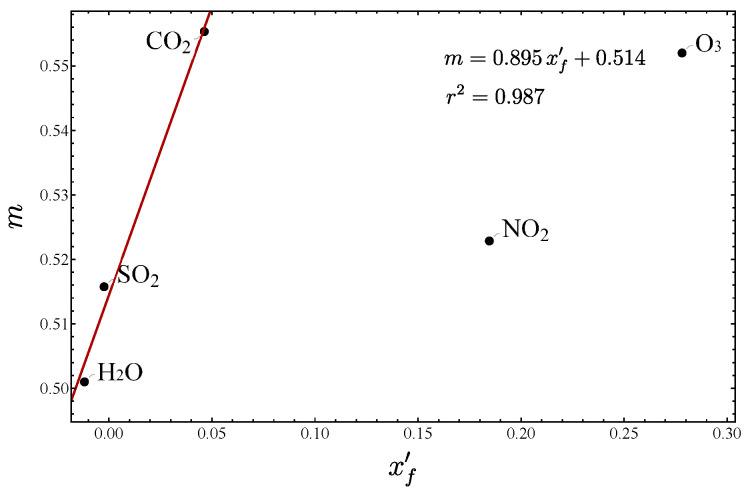
Slope *m* vs. $x_{f}^{\prime }$ for the isotopologues associated with the molecules H${}_{2}$O, SO${}_{2}$, CO${}_{2}$, O${}_{3}$, and NO${}_{2}$. The fundamentals for nitrogen dioxide were obtained from Refs. [59,60].

The case of equivalent oscillators embraces the isotopologues of type ${}^{x}A^{y}B_{2}$. Examples of molecules with sufficient experimental information include $H_{2}O$, NO${}_{2}$, SO${}_{2}$, O${}_{3}$, and CO${}_{2}$. In Figure 5, the plots $\delta_{-}$ vs. γ are displayed for $H_{2}O$, SO${}_{2}$, and O${}_{3}$. For the isotopologues of carbon dioxide, the corresponding plot is given in Figure 1 of Ref. [33]. For NO${}_{2}$, only two isotopologues are given, and no plot is necessary. In Figure 6, the force constants vs. the slope *m* are depicted. The molecules $H_{2}O$, SO${}_{2}$, and CO${}_{2}$ are in close agreement with the expected linear trend, although for SO${}_{2}$ and CO${}_{2}$, we have corrected the slope, adding higher order terms to $\gamma^{\left(\omega \right)}$, as explained before. However, two molecules, NO${}_{2}$ and O${}_{3}$, present a quite different behavior, which we believe is due to their resonant structure manifested by the different values of force constants.

In this analysis, our results are obtained either using force constants from ab initio calculations or from the fundamentals, but in either case the force constants must be the same for every isotopologue.

## 4. Pyramidal Molecules

In Ref. [20], the case of three equivalent oscillators was analyzed in the context of the pyramidal molecules for both stretching and bending modes, while in Ref. [61] a study of the stretching modes in the molecule BF${}_{3}$ was considered. Here, we focus on pyramidal molecules, because of their abundance.

Pyramidal molecules present two stretching and two bending normal modes, both with symmetries $A_{1}⊕E$. The fundamental energies of the bending modes are far from the stretching frequencies, hence a good approximation consists in neglecting stretching–bending interactions up to second order, where the normal modes are defined. This means that the stretches and bends can be independently analyzed in terms of a Hamiltonian of type (2).

We start with the stretching degrees of freedom. The expressions for the force constants (3) are [20]

(48a) $$\begin{array}{ccc} & F_{A_{1}A_{1}}=f_{rr}+2f_{rr^{\prime }};\; & G_{A_{1}A_{1}}^{\left(0\right)}=g_{rr}^{\left(0\right)}+2g_{rr^{\prime }}^{\left(0\right)}, \end{array}$$

(48b) $$\begin{array}{ccc} & F_{EE}=f_{rr}-f_{rr^{\prime }};\; & G_{EE}^{\left(0\right)}=g_{rr}^{\left(0\right)}-g_{rr^{\prime }}^{\left(0\right)}, \end{array}$$

while for the matrix elements involved in (6)

(49) $$\left|\right|m_{i,\Gamma \gamma }\left|\right|=\left(\begin{array}{ccc} \frac{1}{\sqrt{3}} & \frac{2}{\sqrt{6}} & 0\\ \frac{1}{\sqrt{3}} & -\frac{1}{\sqrt{6}} & \frac{1}{\sqrt{2}}\\ \frac{1}{\sqrt{3}} & -\frac{1}{\sqrt{6}} & -\frac{1}{\sqrt{2}} \end{array}\right),$$

which was chosen to be associated with the group chain $C_{3v}⊃C_{s}^{a}$ with $C_{s}^{a}=\left\{E,\sigma_{v}^{a}\right\}$, with the same notation for the symmetry elements used in Ref. [20]. In this case, the algebraic Hamiltonian (8) takes the form

(50) $$\begin{array}{c} {\hat{H}}_{L}=\lambda_{0}\sum_{i=1}^{3}\left({\hat{a}}_{i}^{†}{\hat{a}}_{i}+{\hat{a}}_{i}{\hat{a}}_{i}^{†}\right)+\lambda_{1}^{\left(1\right)}\sum_{i>j=1}^{3}\left({\hat{a}}_{i}^{†}{\hat{a}}_{j}+{\hat{a}}_{i}{\hat{a}}_{j}^{†}\right)+\lambda_{2}^{\left(1\right)}\sum_{i>j=1}^{3}\left({\hat{a}}_{i}^{†}{\hat{a}}_{j}^{†}+{\hat{a}}_{i}{\hat{a}}_{j}\right), \end{array}$$

with coefficients given by (11). This Hamiltonian does not conserve the local polyad. Again, if we demand $\lambda_{2}^{\left(1\right)}=0$, we are able to estimate the force constants using (11), as well as the matrix representation of the Hamiltonian in the local basis L={|100〉,|010〉,|001〉}. The results were extracted from Table 1 with $\mu_{2}=E$, allowing the parameters $\epsilon^{\prime }s$ to be calculated.

To obtain a polyad-conserving Hamiltonian, we should consider the canonical transformation (15), which when substituted into the Hamiltonian (2) yields

(51) $${\hat{H}}_{L^{\prime }}^{\left(P_{L}\right)}=\omega_{nor}\sum_{i=1}^{3}\left({\hat{c}}_{i}^{†}{\hat{c}}_{i}+{\hat{c}}_{i}{\hat{c}}_{i}^{†}\right)+\lambda_{nor}^{\left(1\right)}\sum_{i>j=1}^{3}\left({\hat{c}}_{i}^{†}{\hat{c}}_{j}+{\hat{c}}_{i}{\hat{c}}_{j}^{†}\right),$$

where the spectroscopic parameters given by

(52a) $$\begin{array}{ccc} \omega_{nor}= & \frac{ћ\omega_{0}}{2} & \left(\;\frac{1}{3}\sqrt{\left(1+2x_{f}^{\prime }\right)\left(1+2x_{g}^{\prime }\right)}+\frac{2}{3}\sqrt{\left(1-x_{f}^{\prime }\right)\left(1-x_{g}^{\prime }\right)}\right), \end{array}$$

(52b) $$\begin{array}{ccc} \lambda_{nor}^{\left(1\right)}= & \frac{ћ\omega_{0}}{2} & \left(\;\frac{2}{3}\sqrt{\left(1+2x_{f}^{\prime }\right)\left(1+2x_{g}^{\prime }\right)}-\frac{2}{3}\sqrt{\left(1-x_{f}^{\prime }\right)\left(1-x_{g}^{\prime }\right)}\;\right). \end{array}$$

are functions of x, in accordance with (18). Their expansion, equivalent to (18), leads to the identification

(53) $$\gamma^{\left(12\right)}\to \gamma^{\left(1\right)}=\gamma^{\left(\omega \right)}=\frac{1}{4}{\left(x_{f}^{\prime }-x_{g}^{\prime }\right)}^{2},$$

which again turns out to be basically the Hessian of the Taylor expansions of (52). In Table 4, the fundamentals as well as the calculated force constants are given for several molecules. Because it is possible to establish an isomorphism between the stretches and the bends in such a way that both span the same irreducible representations, the general results are basically valid, with the proviso that the structure constants change for the bends [20]. In Table 5, the LN parameters for the stretches are presented, while in Table 6 the corresponding parameters for the bends are listed.

**Figure 7:**
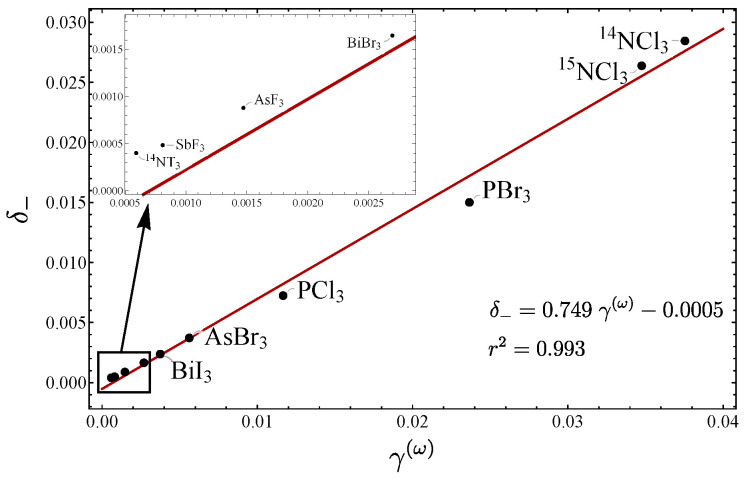
Plot of $\delta_{-}$ vs. $\gamma^{\left(\omega \right)}$ for the stretching modes of pyramidal molecules.

**Figure 8:**
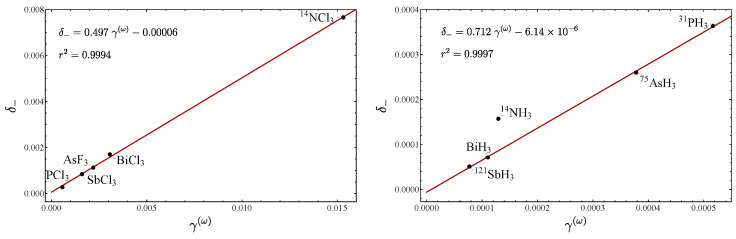
Plots of $\delta_{-}$ vs. $\gamma^{\left(\omega \right)}$ for the bending modes of pyramidal molecules.

In Figure 7, a plot of $\delta_{-}$ vs. $\gamma^{\left(\omega \right)}$ is displayed for the stretching modes for several molecules, including the pyramidal molecules analyzed in Ref. [20]. Again, a linear trend is manifested, because all the molecules are in the local mode region (small values of $\delta_{-}$). In Figure 8, the plot $\delta_{-}$ vs. $\gamma^{\left(\omega \right)}$ for the bending modes is displayed. One point to stress is that the NH${}_{3}$ molecule is the only one located out of the line, a fact we assumed to be due to the existence of the inversion mode [20]. We have thus identified two cases where the deviation of the linear behavior allows particular molecular properties to be identified: internal inversion and resonance.

Although we do not present the plot $\epsilon_{1}$ vs. $\delta_{-}$, it turned out to be similar for both stretches and bends, a behavior that can be extracted from Table 5 and Table 6. Regarding $\epsilon_{2}$, for the stretches, the expected correlation with $\delta_{-}$ is fulfilled, while this is not the case for the bends. From the same Tables, it is clear that the parameter ζ associated with the splitting of labels does not represent an LN parameter.

## 5. Tetrahedral Molecules

Here, we present a study of tetrahedral molecules for both stretching and bending degrees of freedom. This system is relevant because the bending modes present a spurious state. First, we consider the four stretching oscillators, which reduce to the $A_{1}⊕F_{2}$ irreducible representations. Again, the fundamental energies of the bending modes are far from the stretching frequencies, and consequently to obtain the normal modes, a good approximation consists in neglecting stretching–bending interactions up to second order.

### 5.1. Stretching Oscillators

The Hamiltonian in the normal scheme for the stretching modes takes the form (2), with

(54a) $$\begin{array}{ccc} & F_{A_{1}A_{1}}=f_{rr}+3f_{rr^{\prime }};\; & G_{A_{1}A_{1}}^{\left(0\right)}=g_{rr}^{\left(0\right)}+3g_{rr^{\prime }}^{\left(0\right)}, \end{array}$$

(54b) $$\begin{array}{ccc} & F_{F_{2}F_{2}}=f_{rr}-f_{rr^{\prime }};\; & G_{F_{2}F_{2}}^{\left(0\right)}=g_{rr}^{\left(0\right)}-g_{rr^{\prime }}^{\left(0\right)}. \end{array}$$

From these expressions and the fundamentals, we obtained the estimation of the force constants (3) given in Table 1 with $\mu_{2}=F_{2}$. Here, the components of $F_{2}$ are labeled in accordance with the canonical chain $T_{d}⊃C_{2v}$. The connection between the normal bosonic operators and the local operators is given by (4), with $\delta_{\pm \Gamma }$ obtained through (5). The matrix elements $\left|\right|m_{i,\Gamma \gamma }\left|\right|$ define the symmetry adapted combinations for one quantum

(55) $$\left|\right|m_{i,\Gamma \gamma }\left|\right|=\left(\begin{array}{cccc} \frac{1}{2} & \frac{1}{2} & \frac{1}{\sqrt{2}} & 0\\ \frac{1}{2} & -\frac{1}{2} & 0 & \frac{1}{\sqrt{2}}\\ \frac{1}{2} & \frac{1}{2} & -\frac{1}{\sqrt{2}} & 0\\ \frac{1}{2} & -\frac{1}{2} & 0 & -\frac{1}{\sqrt{2}} \end{array}\right)$$

with order $\left\{A_{1},\left(F_{2},A_{1}\right),\left(F_{2},B_{2}\right),\left(F_{2},B_{3}\right)\right\}$.

The substitution of (4) into the Hamiltonian (2) leads to the algebraic representation of the Hamiltonian in the local scheme

(56) $$\begin{array}{c} {\hat{H}}_{L}=\lambda_{0}\sum_{i=1}^{4}\left({\hat{a}}_{i}^{†}{\hat{a}}_{i}+{\hat{a}}_{i}{\hat{a}}_{i}^{†}\right)+\lambda_{1}^{\left(1\right)}\sum_{i>j=1}^{4}\left({\hat{a}}_{i}^{†}{\hat{a}}_{j}+{\hat{a}}_{i}{\hat{a}}_{j}^{†}\right)+\lambda_{2}^{\left(1\right)}\sum_{i>j=1}^{4}\left({\hat{a}}_{i}^{†}{\hat{a}}_{j}^{†}+{\hat{a}}_{i}{\hat{a}}_{j}\right), \end{array}$$

with coefficients defined by (11) with the convention $\lambda^{\left(12\right)}\to \lambda^{\left(1\right)}$, since only one type of interaction is present. As expected, this Hamiltonian does not conserve the local polyad. Demanding $\lambda_{2}^{\left(1\right)}=0$ in (56) and taking its diagonalization in the space of one local quanta L={|1000〉,|0100〉,|0010〉,|0001〉}, we are able to estimate the force constants $f_{rr}^{\left(L\right)},f_{rr^{\prime }}^{\left(L\right)}$ from Table 1.

Let us now consider the equivalent expression (16). The normal operators neglecting the contribution of the annihilation local operators are given by (15). Their substitution into the Hamiltonian (2) leads to

(57) $${\hat{H}}_{L^{\prime }}^{\left(P_{L}\right)}=\omega_{nor}\sum_{i=1}^{4}\left({\hat{c}}_{i}^{†}{\hat{c}}_{i}+{\hat{c}}_{i}{\hat{c}}_{i}^{†}\right)+\lambda_{nor}^{\left(1\right)}\sum_{i>j=1}^{4}\left({\hat{c}}_{i}^{†}{\hat{c}}_{j}+{\hat{c}}_{i}{\hat{c}}_{j}^{†}\right),$$

with coefficients

(58a) $$\begin{array}{ccc} \omega_{nor}= & \frac{ћ\omega_{0}}{2} & \left(\frac{1}{4}\sqrt{\left(1+x_{f}^{\prime }\right)\left(1+3x_{g}^{\prime }\right)}+\frac{3}{4}\sqrt{\left(1-x_{f}^{\prime }\right)\left(1-x_{g}^{\prime }\right)}\right), \end{array}$$

(58b) $$\begin{array}{ccc} \lambda_{nor}^{\left(1\right)}= & \frac{ћ\omega_{0}}{2} & \left(\;\frac{1}{2}\sqrt{\left(1+3x_{f}^{\prime }\right)\left(1+3x_{g}^{\prime }\right)}-\frac{1}{2}\sqrt{\left(1-x_{f}^{\prime }\right)\left(1-x_{g}^{\prime }\right)}\;\right), \end{array}$$

which when expanded in terms of the $x_{f}^{\prime }$ and $x_{g}^{\prime }$ allow the identification

(59a) $$\begin{array}{ccc} \gamma^{\left(\omega \right)} & = & \frac{3}{8}{\left(x_{f}^{\prime }-x_{g}^{\prime }\right)}^{2}; \end{array}$$

(59b) $$\begin{array}{ccc} \gamma^{\left(12\right)}\to \gamma^{\left(1\right)} & = & \frac{1}{2}{\left(x_{f}^{\prime }-x_{g}^{\prime }\right)}^{2}. \end{array}$$

Since these expressions are proportional, we introduce the unique parameter

(60) $$\gamma \equiv {\left(x_{f}^{\prime }-x_{g}^{\prime }\right)}^{2}.$$

The fits of the parameters involved in the Hamiltonians (56) and (57) to reproduce the fundamentals. Both fits provide the same numerical values for the sets $\left\{\lambda_{0},\lambda_{1}^{\left(1\right)}\right\}$ and $\left\{\omega_{nor},\lambda_{nor}^{\left(1\right)}\right\}$, but the connections to the force constants are different. From these results the parameters $\epsilon^{\prime }s$ can be obtained.

**Figure 9:**
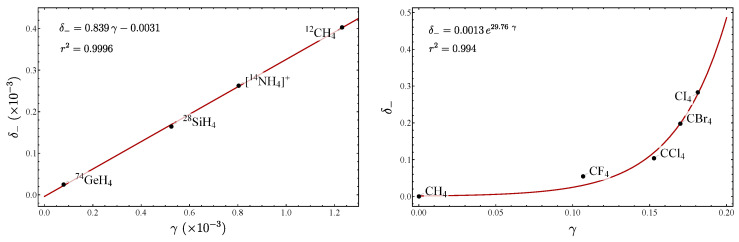
Plot of $\delta_{-}$ vs. γ for the stretching degrees of freedom of tetrahedral molecules. On the right, the exponential form is manifested due to the high degree of normality of the molecules.

In Table 7, the fundamentals and the force constants calculated from Table 1 are given, while in Table 8 the LN parameters can be found. Using these results in Figure 9 the plot of $\delta_{-}$ vs. γ is depicted, including several tetrahedral molecules for the stretching modes. On the right side of the figure, molecules with extreme normal mode behavior are displayed, while on the left, molecules with local mode behavior are included. The exponential trend in the former, behavior explained in Section 3.3.

### 5.2. Bending Oscillators

The set of bending oscillators in tetrahedral molecules is interesting because of the presence of a spurious state, a common situation in molecules with high symmetry in the framework of internal coordinates. The subspace of six oscillators spans the irreducible representations $A_{1}⊕E⊕F_{2}$, with $A_{1}$ identified as a spurious mode. Consequently, the Hamiltonian (2) only involves the $E⊕F_{2}$ modes with

(61a) $$\begin{array}{ccc} \Omega_{E} & = & \sqrt{\left(f_{\theta \theta }-2f_{\theta \theta^{\prime }}+f_{\theta \theta^{\prime \prime }}\right)\left(g_{\theta \theta }^{\left(0\right)}-2g_{\theta \theta^{\prime }}^{\left(0\right)}+g_{\theta \theta^{\prime \prime }}^{\left(0\right)}\right)} \end{array}$$

(61b) $$\begin{array}{ccc} \Omega_{F_{2}} & = & \sqrt{\left(f_{\theta \theta }-f_{\theta \theta^{\prime }}\right)\left(g_{\theta \theta }^{\left(0\right)}-g_{\theta \theta^{\prime }}^{\left(0\right)}\right)}. \end{array}$$

Here, we have three force constants and two fundamentals with the constraint

(62) $$\Omega_{A_{1}}=\sqrt{\left(f_{\theta \theta }+4f_{\theta \theta^{\prime }}+f_{\theta \theta^{\prime \prime }}\right)\left(g_{\theta \theta }^{\left(0\right)}+4g_{\theta \theta^{\prime }}^{\left(0\right)}+g_{\theta \theta^{\prime \prime }}^{\left(0\right)}\right)}=0.$$

The estimation of the force constants are given in Table 1, with $\mu_{2}=E$ and $\mu_{3}=F_{2}$, omitting from the sums the spurious state $A_{1}$. The normal and the local operators are related through the transformation (4). The symmetry projection matrix takes the form

(63) ||mi,Γγ||=16−13−0−12−0−016−123−12−0−12−1216−123−12−0−12−1216−13−0−12−0−016−123−12−0−12−1216−123−12−0−12−12

Here, the projection is associated with the group chain $T_{d}⊃C_{2v}$ with the following order for the irreducible representations $\left\{A_{1},\left(E,A_{1}\right),\left(E,A_{2}\right),\left(F_{2},A_{1}\right),\left(F_{2},B_{1}\right),\left(F_{2},B_{2}\right)\right\}$.

Because of the presence of the spurious mode, this system will be analyzed following a different route. The substitution of the transformations (4) into (2), without eliminating the spurious state $A_{1}$, leads to the local representation

(64) $$\begin{array}{ccc} {\hat{H}}_{L} & = & \lambda_{0}\sum_{i}\left({\hat{a}}_{i}^{†}{\hat{a}}_{i}+{\hat{a}}_{i}{\hat{a}}_{i}^{†}\right)+\lambda_{1}^{\left(1\right)}\sum_{j>i}\left({\hat{a}}_{i}^{†}{\hat{a}}_{j}+{\hat{a}}_{i}{\hat{a}}_{j}^{†}\right)+\lambda_{1}^{\left(2\right)}\sum_{j^{\prime }>i}\left({\hat{a}}_{i}^{†}{\hat{a}}_{j^{\prime }}{\hat{a}}_{i}{\hat{a}}_{j^{\prime }}^{†}\right)\\ & + & \lambda_{2}^{\left(1\right)}\sum_{j>i}\left({\hat{a}}_{i}^{†}{\hat{a}}_{j}^{†}+{\hat{a}}_{i}{\hat{a}}_{j}\right)+\lambda_{2}^{\left(2\right)}\sum_{j^{\prime }>i}\left({\hat{a}}_{i}^{†}{\hat{a}}_{j^{\prime }}^{†}+{\hat{a}}_{i}{\hat{a}}_{j^{\prime }}\right), \end{array}$$

with coefficients

(65a) $$\begin{array}{ccc} \lambda_{0} & = & ћ\left[\frac{\Omega_{A_{1}}}{12}\left(\delta_{+,A_{1}}+\delta_{-,A_{1}}\right)+\frac{\Omega_{E}}{6}\left(\delta_{+,E}+\delta_{-,E}\right)+\frac{\Omega_{F_{2}}}{4}\left(\delta_{+,F_{2}}+\delta_{-,F_{2}}\right)\right]\;; \end{array}$$

(65b) $$\begin{array}{ccc} \lambda_{1}^{\left(1\right)} & = & ћ\left[\frac{\Omega_{A_{1}}}{12}\left(\delta_{+,A_{1}}+\delta_{-,A_{1}}\right)-\frac{\Omega_{E}}{12}\left(\delta_{+,E}+\delta_{-,E}\right)\right]\;; \end{array}$$

(65c) $$\begin{array}{ccc} \lambda_{1}^{\left(2\right)} & = & ћ\left[\frac{\Omega_{A_{1}}}{12}\left(\delta_{+,A_{1}}+\delta_{-,A_{1}}\right)+\frac{\Omega_{E}}{12}\left(\delta_{+,E}+\delta_{-,E}\right)-\frac{\Omega_{F_{2}}}{8}\left(\delta_{+,F_{2}}+\delta_{-,F_{2}}\right)\right]\;; \end{array}$$

(65d) $$\begin{array}{ccc} \lambda_{2}^{\left(1\right)} & = & ћ\left[\frac{\Omega_{A_{1}}}{12}\sqrt{\delta_{+,A_{1}}\delta_{-,A_{1}}}-\frac{\Omega_{E}}{12}\sqrt{\delta_{+,E}\delta_{-,E}}\right]\;; \end{array}$$

(65e) $$\begin{array}{ccc} \lambda_{2}^{\left(2\right)} & = & ћ\left[\frac{\Omega_{A_{1}}}{12}\sqrt{\delta_{+,A_{1}}\delta_{-,A_{1}}}+\frac{2\Omega_{E}}{12}\;\sqrt{\delta_{+,E}\delta_{-,E}}-\frac{\Omega_{F_{2}}}{4}\;\sqrt{\delta_{+,F_{2}}\delta_{-,F_{2}}}\right],\; \end{array}$$

where here we notice that two types of pairwise interactions appear. We decided to write down the coefficients in this form, because this makes evident that when $\delta_{-\Gamma }=0$, we obtain the expected results (11). However, when the $A_{1}$ mode is eliminated, the coefficients become

(66a) $$\begin{array}{ccc} \lambda_{0} & = & -\frac{\omega_{0}}{24}\left[-10+4\left(x_{f}^{\prime }+x_{g}^{\prime }\right)+\left(x_{f}^{\prime \prime }+x_{g}^{\prime \prime }\right)\right] \end{array}$$

(66b) $$\begin{array}{ccc} \lambda_{1}^{\left(1\right)} & = & -\frac{\omega_{0}}{24}\left[2-2\left(x_{f}^{\prime }+x_{g}^{\prime }\right)+\left(x_{f}^{\prime \prime }+x_{g}^{\prime \prime }\right)\right], \end{array}$$

(66c) $$\begin{array}{ccc} \lambda_{1}^{\left(2\right)} & = & -\frac{\omega_{0}}{24}\left[2+4\left(x_{f}^{\prime }+x_{g}^{\prime }\right)-5\left(x_{f}^{\prime \prime }+x_{g}^{\prime \prime }\right)\right], \end{array}$$

(66d) $$\begin{array}{ccc} \lambda_{2}^{\left(1\right)} & = & -\frac{\omega_{0}}{24}\left[-2\left(x_{f}^{\prime }-x_{g}^{\prime }\right)+\left(x_{f}^{\prime \prime }-x_{g}^{\prime \prime }\right)\right], \end{array}$$

(66e) $$\begin{array}{ccc} \lambda_{2}^{\left(2\right)} & = & -\frac{\omega_{0}}{24}\left[4\left(x_{f}^{\prime }-x_{g}^{\prime }\right)-5\left(x_{f}^{\prime \prime }-x_{g}^{\prime \prime }\right)\right]. \end{array}$$

As noticed, the general form (11) has been broken. But we will not deal with the expressions (66) used to calculate the force constants. Instead, we return to (11), as we next explain. The simplest way to proceed consists in starting with the Hamiltonian (64), but neglecting the non-conserving polyad contributions:

(67) $$\begin{array}{c} {\hat{H}}_{L}^{\left(P_{L}\right)}=\lambda_{0}\sum_{i}\left({\hat{a}}_{i}^{†}{\hat{a}}_{i}+{\hat{a}}_{i}{\hat{a}}_{i}^{†}\right)+\lambda_{1}^{\left(1\right)}\sum_{i\neq j}\left({\hat{a}}_{i}^{†}{\hat{a}}_{j}+{\hat{a}}_{i}{\hat{a}}_{j}^{†}\right)+\lambda_{1}^{\left(2\right)}\sum_{i\neq j^{\prime }}\left({\hat{a}}_{i}^{†}{\hat{a}}_{j^{\prime }}+{\hat{a}}_{i}\;{\hat{a}}_{j^{\prime }}^{†}\right)\;, \end{array}$$

with the identification (11). The diagonalization of this Hamiltonian in one quantum local basis $L_{6}=\{|100000\rangle ,|010000\rangle ,|001000\rangle ,|000100\rangle ,|000010\rangle ,|000001\rangle ,\}$ leads to three eigenvalues, which when they are identified with the fundamentals $ћ\Omega_{\Gamma }$ lead to

(68a) $$\begin{array}{ccc} ћ\Omega_{A_{1}} & = & ћ\omega_{0}\left[1+2\left(x_{f}^{\prime }+x_{g}^{\prime }\right)+\frac{1}{2}\left(x_{f}^{\prime \prime }+x_{g}^{\prime \prime }\right)\right] \end{array}$$

(68b) $$\begin{array}{ccc} ћ\Omega_{E} & = & ћ\omega_{0}\left[1-\left(x_{f}^{\prime }+x_{g}^{\prime }\right)+\frac{1}{2}\left(x_{f}^{\prime \prime }+x_{g}^{\prime \prime }\right)\right] \end{array}$$

(68c) $$\begin{array}{ccc} ћ\Omega_{F_{2}} & = & ћ\omega_{0}\left[1-\frac{1}{2}\left(x_{f}^{\prime \prime }+x_{g}^{\prime \prime }\right)\right], \end{array}$$

When we impose the condition (62) with definitions (12), we are able to obtain the local force constants $f_{q_{i}q_{j}}^{\left(L\right)}$ from the equations given in Table 1.

We now proceed to obtain the $\gamma^{\prime }s$ parameters. Applying the transformation (15) to the Hamiltonian (2), preserving the spurious state, we obtain

(69) $$\begin{array}{c} {\hat{H}}_{L^{\prime }}^{\left(P_{L}\right)}=\omega_{nor}\sum_{i}\left({\hat{c}}_{i}^{†}{\hat{c}}_{i}+{\hat{c}}_{i}{\hat{c}}_{i}^{†}\right)+\lambda_{nor}^{\left(1\right)}\sum_{i\neq j}\left({\hat{c}}_{i}^{†}{\hat{c}}_{j}+{\hat{c}}_{i}{\hat{c}}_{j}^{†}\right)+\lambda_{nor}^{\left(2\right)}\sum_{i\neq j^{\prime }}\left({\hat{c}}_{i}^{†}{\hat{c}}_{j^{\prime }}+{\hat{c}}_{i}{\hat{c}}_{j^{\prime }}^{†}\right)\;, \end{array}$$

where

(70a) $$\begin{array}{ccc} \omega_{nor} & = & ћ\left(\frac{\Omega_{A_{1}}}{12}+\frac{\Omega_{E}}{6}+\frac{\Omega_{F_{2}}}{4}\right)\;; \end{array}$$

(70b) $$\begin{array}{ccc} \lambda_{nor}^{\left(1\right)} & = & ћ\left(\frac{\Omega_{A_{1}}}{12}-\frac{\Omega_{E}}{12}\right)\;; \end{array}$$

(70c) $$\begin{array}{ccc} \lambda_{nor}^{\left(2\right)} & = & ћ\left(\frac{\Omega_{A_{1}}}{12}+\frac{\Omega_{E}}{6}-\frac{\Omega_{F_{2}}}{4}\right)\;, \end{array}$$

The Equations (70) are implicit functions of the variables x. Their expansion leads to

(71) $$\begin{array}{ccc} \omega_{nor} & = & ћ\omega_{0}\left[\frac{1}{2}-\gamma^{\left(\omega \right)}+O\left(x^{3}\right)\right]\;; \end{array}$$

(72) $$\begin{array}{ccc} \lambda_{nor}^{\left(1\right)} & = & ћ\omega_{0}\left[\frac{1}{4}\left(x_{f}^{\prime }+x_{g}^{\prime }\right)-\gamma^{\left(1\right)}+O\left(x^{3}\right)\right]\;; \end{array}$$

(73) $$\begin{array}{ccc} \lambda_{nor}^{\left(2\right)} & = & ћ\omega_{0}\left[\frac{1}{4}\left(x_{f}^{\prime \prime }+x_{g}^{\prime \prime }\right)-\gamma^{\left(2\right)}+O\left(x^{3}\right)\right]\;, \end{array}$$

with the identification

(74a) $$\begin{array}{ccc} \gamma^{\left(\omega \right)} & = & \frac{1}{4}{\left(x_{f}^{\prime }-x_{g}^{\prime }\right)}^{2}+\frac{1}{16}{\left(x_{f}^{\prime \prime }-x_{g}^{\prime \prime }\right)}^{2}\;; \end{array}$$

(74b) $$\begin{array}{ccc} \gamma^{\left(1\right)} & = & \frac{1}{8}\left(x_{f}^{\prime }-x_{g}^{\prime }\right)\left[\left(x_{f}^{\prime }-x_{g}^{\prime }\right)+\left(x_{f}^{\prime \prime }-x_{g}^{\prime \prime }\right)\right]\;; \end{array}$$

(74c) $$\begin{array}{ccc} \gamma^{\left(2\right)} & = & \frac{1}{4}{\left(x_{f}^{\prime }-x_{g}^{\prime }\right)}^{2}\;. \end{array}$$

**Figure 10:**
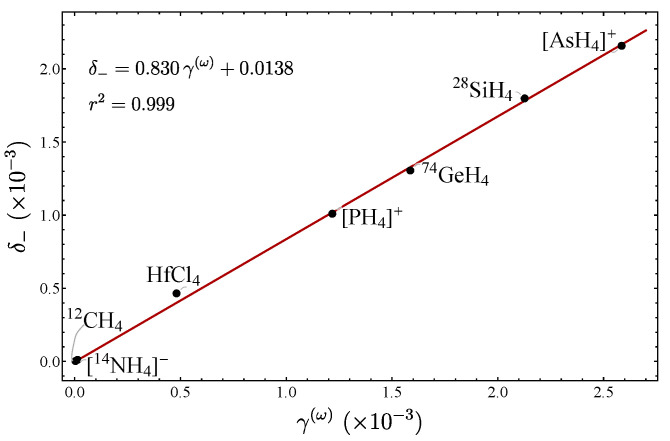
Plot of $\delta_{-}$ vs. $\gamma^{\left(\omega \right)}$ for tetrahedral molecules involving the bending degrees of freedom.

**Figure 11:**
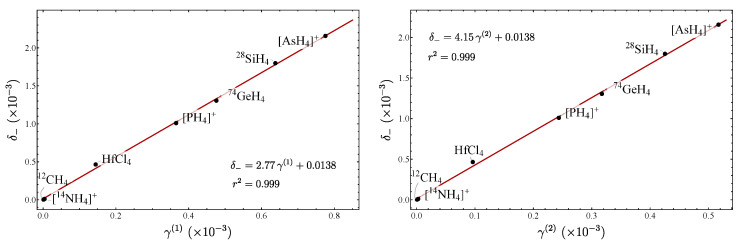
Plot of $\delta_{-}$ vs. $\gamma^{\left(1\right)}$ and $\delta_{-}$ vs. $\gamma^{\left(2\right)}$ for tetrahedral molecules involving the bending degrees of freedom.

We stress that in these expressions the spurious state $A_{1}$ has been included. However, the redundancy takes into account the calculation of force constants $f_{q_{i}q_{j}}^{\left(N\right)}$ through the use of (61) together with the constraint (62). In Table 9, the LN parameters are listed, while in Figure 10, the plot $\delta_{-}$ vs. $\gamma^{\left(\omega \right)}$ is displayed. In addition in Figure 11, the corresponding plots involving the parameters $\gamma^{\left(1\right)}$ and $\gamma^{\left(2\right)}$ are presented. These results show the consistency of the parameters in establishing an LN degree, even in the presence of spurious states.

### 5.3. Isotopologues

**Figure 12:**
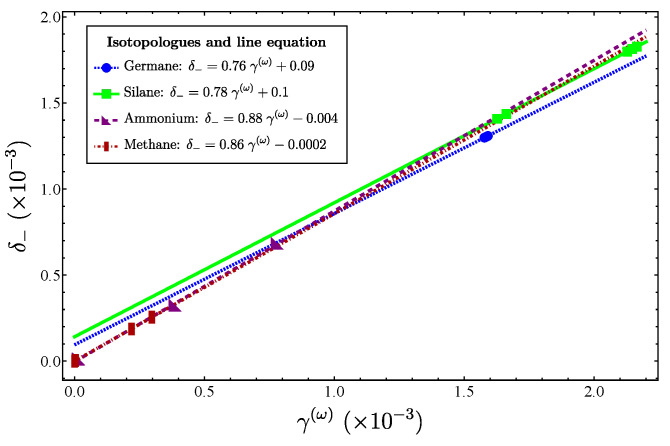
Plot of $\delta_{-}$ vs. $\gamma^{\left(\omega \right)}$ for the different series of isotopologues.

Let us now consider the series of isotopologues ${}^{\alpha }$X${}^{\beta }$H${}_{4}$, X = Ge, Si, N, C; involving the bending degrees of freedom. The plots $\delta_{-}$ vs. $\gamma^{\left(\omega \right)}$ are displayed in Figure 12. Since similar results were obtained for $\delta_{-}$ vs. $\gamma^{\left(1\right)}$ and $\delta_{-}$ vs. $\gamma^{\left(2\right)}$, their corresponding plots are not included. The linear correlations are evident, as in the previous cases. This is clearly explained by the local mode behavior of the systems. However, this is not an obvious result, since we are dealing with bending degrees of freedom presenting a spurious state. We again stress that bending modes are not traditionally contemplated when assigning an LN degree, but with these results we confirm that the parameters we have introduced represent a valid measure of the LN degree.

**Figure 13:**
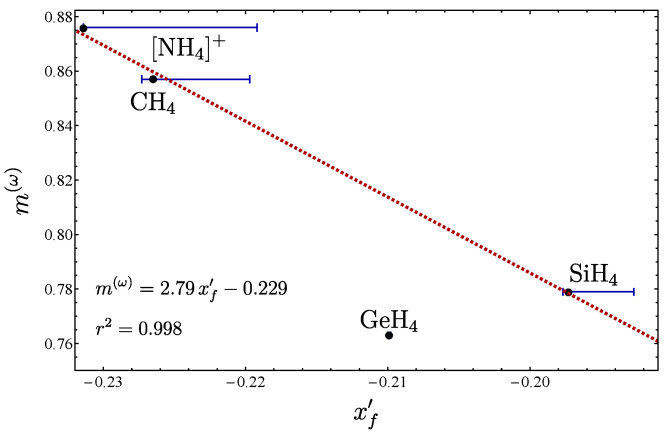
Plot of $m^{\left(\omega \right)}$ vs. $x_{f}^{\prime }$ associated with the isotopologues of Figure 12. [NH_4_${]}^{+}$, CH_4_, and SiH_4_ lie along the line, while GeH_4_ is manifested outside of it.

From Figure 12, we may also consider the slopes to look for a correlation with the force constants $x_{f}^{\prime }$. The result is presented in Figure 13. In order to create the plot, we had to take into account that several force constants are available. This fact is taken into account by the bars in the plot. We can see that with the exception of GeH${}_{4}$, a line can be assigned for the correlation. We believe that the odd behavior of GeH${}_{4}$ with respect to the other compounds is due to the Germanium configuration $\left[Ar\right]4s^{2}3d^{10}4p^{2}$, in contrast to C, N, and Si, where no *d* orbitals are present. A similar situation appears in the series of pyramidal molecules, where the unexpected local-to-normal order is believed to have the same origin [20].

## 6. Octahedral Molecules

We now consider the stretching modes of octahedral molecules, with the $O_{h}$ symmetry group. This system is included in our study because of the presence of two types of interactions: contiguous and opposite ones, but also because, in the case of contiguous bonds, the parameter $x_{g}^{\prime }$ vanishes. A partial analysis of this system, including vibrational descriptions of the series of molecules SF${}_{6}$, WF${}_{6}$, and UF${}_{6}$, was discussed in Ref. [32].

The reduction of the internal coordinates corresponds to $A_{1g}⊕E_{g}⊕T_{1u}$. In this case, the force and structure constants take the form

(75a) $$\begin{array}{ccc} & F_{A_{1g}A_{1g}}=f_{rr}+4f_{rr^{\prime }}+f_{rr^{\prime \prime }};\; & G_{A_{1g}A_{1g}}^{\left(0\right)}=g_{rr}^{\left(0\right)}+4g_{rr^{\prime }}^{\left(0\right)}+g_{rr^{\prime \prime }}^{\left(0\right)}; \end{array}$$

(75b) $$\begin{array}{ccc} & F_{E_{g}E_{g}}=f_{rr}-2f_{rr^{\prime }}+f_{rr^{\prime \prime }};\; & G_{E_{g}E_{g}}^{\left(0\right)}=g_{rr}^{\left(0\right)}-2g_{rr^{\prime }}^{\left(0\right)}+g_{rr^{\prime \prime }}^{\left(0\right)}; \end{array}$$

(75c) $$\begin{array}{ccc} & F_{T_{1u}T_{1u}}=f_{rr}-f_{rr^{\prime \prime }};\; & G_{T_{1u}T_{1u}}^{\left(0\right)}=g_{rr}^{\left(0\right)}-g_{rr^{\prime \prime }}^{\left(0\right)}. \end{array}$$

from these expressions and the fundamental energies, the force constants can be obtained from Table 1, with $\mu_{3}=T_{1u}$. The results are displayed in Table 10. The matrix elements $\left|\right|m_{i,\Gamma \gamma }\left|\right|$ defining the symmetry-adapted combinations for one quantum are given by

(76) $$\left|\right|m_{i,\Gamma \gamma }\left|\right|=\left(\begin{array}{cccccc} \frac{1}{\sqrt{6}} & \frac{1}{2\sqrt{3}} & \frac{1}{2} & 0 & \frac{1}{2} & \frac{1}{2}\\ \frac{1}{\sqrt{6}} & \frac{1}{2\sqrt{3}} & -\frac{1}{2} & 0 & \frac{1}{2} & -\frac{1}{2}\\ \frac{1}{\sqrt{6}} & \frac{1}{2\sqrt{3}} & \frac{1}{2} & 0 & -\frac{1}{2} & -\frac{1}{2}\\ \frac{1}{\sqrt{6}} & \frac{1}{2\sqrt{3}} & -\frac{1}{2} & 0 & -\frac{1}{2} & \frac{1}{2}\\ \frac{1}{\sqrt{6}} & -\frac{1}{\sqrt{3}} & 0 & \frac{1}{\sqrt{2}} & 0 & 0\\ \frac{1}{\sqrt{6}} & -\frac{1}{\sqrt{3}} & 0 & -\frac{1}{\sqrt{2}} & 0 & 0 \end{array}\right),$$

in accordance with chain $O_{h}⊃D_{2h}$. The substitution of (4) into the Hamiltonian (2) leads to the local representation

(77) $$\begin{array}{ccc} {\hat{H}}_{L} & = & \lambda_{0}\sum_{i=1}^{6}\left({\hat{a}}_{i}^{†}{\hat{a}}_{i}+{\hat{a}}_{i}{\hat{a}}_{i}^{†}\right)+\lambda_{1}^{\left(1\right)}\sum_{i>j}\left({\hat{a}}_{i}^{†}{\hat{a}}_{j}+{\hat{a}}_{i}{\hat{a}}_{j}^{†}\right)+\lambda_{1}^{\left(2\right)}\sum_{i>j^{\prime }}\left({\hat{a}}_{i}^{†}{\hat{a}}_{j^{\prime }}+{\hat{a}}_{i}{\hat{a}}_{j^{\prime }}^{†}\right)\\ & + & \lambda_{2}^{\left(1\right)}\sum_{i>j}\left({\hat{a}}_{i}^{†}{\hat{a}}_{j}^{†}{\hat{a}}_{i}{\hat{a}}_{j}\right)\lambda_{2}^{\left(2\right)}\sum_{i>j^{\prime }}\left({\hat{a}}_{i}^{†}{\hat{a}}_{j^{\prime }}^{†}+{\hat{a}}_{i}{\hat{a}}_{j^{\prime }}\right), \end{array}$$

with coefficients provided by (11). A polyad-conserving Hamiltonian is obtained by setting $\lambda_{2}^{\left(1\right)}=\lambda_{2}^{\left(2\right)}=0$. The diagonalization of the Hamiltonian in the one-quantum basis $L_{6}=\{|100000\rangle ,|010000\rangle ,|001000\rangle ,|000100\rangle ,|000010\rangle ,|000001\rangle \}$ allows us to express the spectroscopic parameters in terms of the fundamental energies, from which we estimate the force constants $f_{q_{i}q_{j}}^{\left(L\right)}$. The results are displayed in Table 1. We may now apply the polyad-conserving transformation (15) to the Hamiltonian (2) to obtain

(78) $$\begin{array}{c} {\hat{H}}_{L^{\prime }}^{\left(P_{L}\right)}=\omega_{nor}\sum_{i=1}^{6}\left({\hat{c}}_{i}^{†}{\hat{c}}_{i}+{\hat{c}}_{i}{\hat{c}}_{i}^{†}\right)+\lambda_{nor}^{\left(1\right)}\sum_{i>j}\left({\hat{c}}_{i}^{†}{\hat{c}}_{j}+{\hat{c}}_{i}{\hat{c}}_{j}^{†}\right)+\lambda_{nor}^{\left(2\right)}\sum_{i>j^{\prime }}\left({\hat{c}}_{i}^{†}{\hat{c}}_{j^{\prime }}+{\hat{c}}_{i}{\hat{c}}_{j^{\prime }}^{†}\right), \end{array}$$

with spectroscopic coefficients given by

(79a) $$\begin{array}{ccc} \omega_{nor} & = & \frac{ћ}{2}\left(\frac{1}{6}\Omega_{A_{1g}}+\frac{1}{3}\Omega_{E_{g}}+\frac{1}{2}\Omega_{T_{1u}}\right), \end{array}$$

(79b) $$\begin{array}{ccc} \lambda_{nor}^{\left(1\right)} & = & \frac{ћ}{2}\left(\frac{1}{3}\Omega_{A_{1g}}-\frac{1}{3}\Omega_{E_{g}}\right), \end{array}$$

(79c) $$\begin{array}{ccc} \lambda_{nor}^{\left(2\right)} & = & \frac{ћ}{2}\left(\frac{1}{3}\Omega_{A_{1g}}+\frac{2}{3}\Omega_{E_{g}}-\Omega_{T_{1u}}\right), \end{array}$$

with

(80a) $$\begin{array}{ccc} \Omega_{A_{1g}} & = & \omega_{0}\sqrt{\left(1+4x_{f}^{\prime }+x_{f}^{\prime \prime }\right)\left(1+4x_{g}^{\prime }+x_{g}^{\prime \prime }\right)} \end{array}$$

(80b) $$\begin{array}{ccc} \Omega_{E_{g}} & = & \omega_{0}\sqrt{\left(1-2x_{f}^{\prime }+x_{f}^{\prime \prime }\right)\left(1-2x_{g}^{\prime }+x_{g}^{\prime \prime }\right)} \end{array}$$

(80c) $$\begin{array}{ccc} \Omega_{T_{1u}} & = & \omega_{0}\sqrt{\left(1-x_{f}^{\prime \prime }\right)\left(1-x_{g}^{\prime \prime }\right)}. \end{array}$$

In this case, the expansion in terms of x leads to the identification

(81a) $$\begin{array}{ccc} \gamma^{\left(\omega \right)} & = & \frac{1}{2}{\left(x_{f}^{\prime }-x_{g}^{\prime }\right)}^{2}+\frac{1}{8}{\left(x_{f}^{\prime \prime }-x_{g}^{\prime \prime }\right)}^{2}\;; \end{array}$$

(81b) $$\begin{array}{ccc} \gamma^{\left(1\right)} & = & \frac{1}{2}\left(x_{f}^{\prime }-x_{g}^{\prime }\right)\left[\left(x_{f}^{\prime }-x_{g}^{\prime }\right)+\left(x_{f}^{\prime \prime }-x_{g}^{\prime \prime }\right)\right]\;; \end{array}$$

(81c) $$\begin{array}{ccc} \gamma^{\left(2\right)} & = & {\left(x_{f}^{\prime }-x_{g}^{\prime }\right)}^{2}\;. \end{array}$$

**Figure 14:**
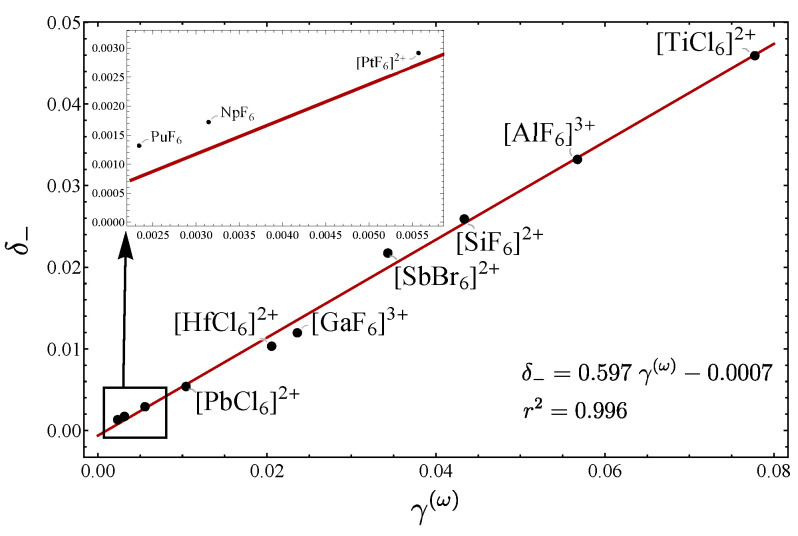
Plot of $\delta_{-}$ vs. $\gamma^{\left(\omega \right)}$ for different octahedral molecules.

**Figure 15:**
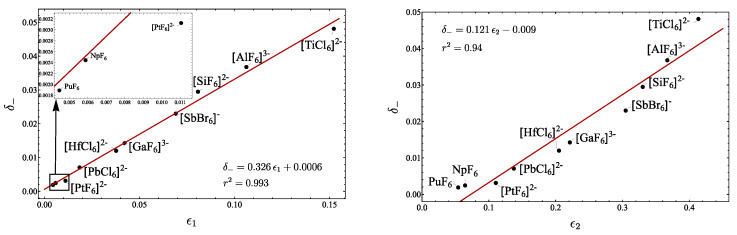
Plots of $\delta_{-}$ vs. $\epsilon_{1}$ and $\delta_{-}$ vs. $\epsilon_{2}$ for different octahedral molecules.

In Table 11, the corresponding parameters associated with the LN degree are presented. Based on these results, in Figure 14 the plot $\delta_{-}$ vs. $\gamma^{\left(\omega \right)}$ is displayed, obtaining a clear linear correlation. In addition, in Figure 15, the plots involving $\epsilon_{1}$ and $\epsilon_{2}$ are included. As expected, the $\epsilon_{1}$ shows a clear linear correlation, while for $\epsilon_{2}$, although a linear trend is obtained, it is not as clear as for $\epsilon_{1}$. The parameter $\epsilon_{3}$ was not included, because no correlation appeared. Again, from Table 11, it is clear that the parameter ζ does not provide a general LN parameter.

## 7. Normal/Local Degree and Physical Properties

The importance of having a parameter measuring the local/normal degree is that it may be correlated with physical properties, depending on the vibrational degrees of freedom. First, we shall consider the effect on the partition function.

### 7.1. Partition Function

**Figure 16:**
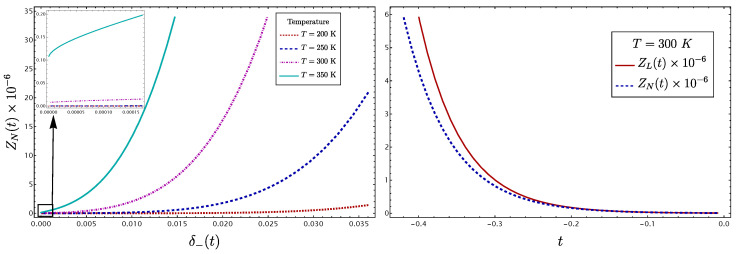
Plots of $Z_{N}\left(t\right)$ at different temperatures, showing the dependence of the LN degree. On the right, the partition function $Z_{L}\left(t\right)$ is shown together with $Z_{N}\left(t\right)$. Both coincide in the local limit, as expected.

As a first case, we shall consider a molecular system in the framework of the Born–Oppenheimer approximation and rotor rigid approximation. If we focus on the stretching vibrational degrees of freedom and consider the Hamiltonian of two equivalent interacting oscillators reduced to the form (2), the partition function $Z_{N}$ takes the simple form

(82) $$Z_{N}=\prod_{\Gamma =g,u}\frac{e^{-\frac{ћ\Omega_{\Gamma }}{2KT}}}{1-e^{-\frac{ћ\Omega_{\Gamma }}{KT}}},$$

with $\Omega_{g}=\omega_{0}\sqrt{\left(1+x_{f}^{\prime }\right)\left(1+x_{g}^{\prime }\right)}$ and $\Omega_{u}=\omega_{0}\sqrt{\left(1-x_{f}^{\prime }\right)\left(1-x_{g}^{\prime }\right)}$. Considering the parameterization (47), which is equivalent to moving along the parameter $\delta_{-}\left(t\right)$ and frequencies $\Omega_{\Gamma }\left(t\right)$, we are able to see the functional form $Z_{N}\left(t\right)$, and consequently the properties depending on it. In Figure 16, left side, the change in the function $Z_{N}\left(t\right)$ along the LN parameter is displayed for different temperatures. We can see that in the local limit the partition function is close to zero and increases as the normal character becomes stronger. On the other hand, for two equivalent oscillators of frequency $\omega_{0}$ without interaction, the partition function takes the form

(83) $$Z_{L}={\left(\frac{e^{-\frac{ћ\omega_{0}}{2KT}}}{1-e^{-\frac{ћ\omega_{0}}{KT}}}\right)}^{2},$$

with $\omega_{0}=\sqrt{f_{rr}g_{rr}^{\left(0\right)}}$. It is clear that in the local limit both coincide: $\lim_{t\to 0}Z_{N}=Z_{L}$. This behavior is shown on the right side of Figure 16. The importance of this analysis is that the dependence on $\delta_{-}$ of the partition function implies a a correlation with thermodynamic properties.

### 7.2. Spectroscopic Properties

We now study the correlation between the parameter $\delta_{-}$ and the wave functions obtained using an algebraic model based on su(2) and su(3) algebras [20,33,53,54]. First, we consider the series of pyramidal molecules PH${}_{3}$, AsH${}_{3}$, and SbH${}_{3}$, which present a local mode behavior. Thereafter, we analyze the isotopologues of carbon dioxide and cyanogen fluoride. The analysis of these systems permits proving that the parameters introduced are correlated with the spectroscopic properties of molecules.

#### 7.2.1. Pyramidal Molecules

Let us start by considering the effect of the LN degree on the wave functions and consequently on the transition intensities. In Ref. [20], a spectroscopic description of the molecules PH${}_{3}$, AsH${}_{3}$, and SbH${}_{3}$ was given. These molecules present a local mode behavior, which is manifested by the locality of the states. In Table 12, the maximum local components for several states characterized by having large local components are displayed. We have chosen states with experimental energies common to the three molecules. The first three states mostly have a stretching character, while the last one has a bending contribution. In order to appreciate the dependence on the LN parameter, the $\delta_{-}$ values have been included. It is interesting to notice the local to normal sequence for the states |200000〉 and |400000〉 is given by SbH${}_{3}\;\to$ PH${}_{3}\;\to$ AsH${}_{3}$, in accordance with the $\delta_{-}$ parameter. In contrast, for the state |200100〉, the sequence changes to SbH${}_{3}\;\to$ AsH${}_{3}\;\to$ PH${}_{3}$. This is explained by the fact that the former set is associated with stretches and the latter to the bends [20]. Hence, we have a correlation between the wave functions and the LN parameter $\delta_{-}$, a result that may also exist in transition intensities.

#### 7.2.2. Isotopologues of CO${}_{2}$

In Refs. [33,53], vibrational analyses were carried out of the series of isotopologues of carbon dioxide using a $SU_{1}\left(2\right)\times SU\left(3\right)\times SU_{2}\left(2\right)$ algebraic model based on the anharmonization of local operators applied to normal operators. It is known that, in these isotopologues, the Fermi interactions dominates the spectrum, a fact manifested in the wave functions. In Figure 3 of Ref. [53], a plot of the Fermi interaction strength $|\alpha_{F}|$ vs. $\delta_{-}$ is displayed, showing a clear correlation. This result suggests that the wave functions will also be correlated. This is indeed the case, as we show next.

In Raman spectroscopy, four of the most intense lines are due to the transitions listed in Table 13 [78]:

These transitions are basically determined by the coefficients *a* and $a^{\prime }$, which turn out to be dominant in the Raman spectrum. The question that arises is whether or not these coefficients are correlated with the parameter $\delta_{-}$. In Table 14, the coefficients of the basis together with the parameter $\delta_{-}$ are displayed. From this table, a correlation is clearly evident and graphically shown in Figure 4 of Ref. [53]. The real importance of this correlation lies in the impact on the description of the Raman spectrum, which may be useful in the identification of isotopologues. Preliminary results show that the Raman transition intensities are indeed correlated with the Raman intensities.

#### 7.2.3. Isotopologues of FCN

As a second example of the importance of the LN degree in vibrational spectroscopy, we consider the vibrational degrees of freedom of the FCN molecule. Given the presence of the resonances $\omega_{1}\approx 2\omega_{2}$ and $2\omega_{1}\approx \omega_{3}$, the appropriate polyad is $P_{N}=2\nu_{1}+2\nu_{2}+4\nu_{3}$. In Ref. [54], vibrational description of this molecule was carried out. Here, we consider the four sets of states corresponding to polyads $P_{N}=2,3,4,5$ associated with symmetries $\Sigma^{+}$ and $\Pi^{\pm }$:

(84a) $$\begin{array}{ccc} \Sigma^{+};P_{N}=2 & : & a|10^{0}0\rangle +b|02^{0}0\rangle , \end{array}$$

(84b) $$\begin{array}{ccc} \Sigma^{+};P_{N}=4 & : & a^{\prime }|04^{0}0\rangle +b^{\prime }|12^{0}0\rangle +c^{\prime }|20^{0}0\rangle +d^{\prime }|00^{0}1\rangle , \end{array}$$

(84c) $$\begin{array}{ccc} \Pi^{\pm };P_{N}=3 & : & \alpha |03^{1}0\rangle +\beta |11^{1}0\rangle , \end{array}$$

(84d) $$\begin{array}{ccc} \Pi^{\pm };P_{N}=5 & : & \alpha^{\prime }|05^{1}0\rangle +\beta^{\prime }|13^{1}0\rangle +\gamma^{\prime }|21^{1}0\rangle +\delta^{\prime }|01^{1}1\rangle . \end{array}$$

For an specific polyad and symmetry, there is a multiplet of states interacting by the two resonances. Hence, the states are characterized by pairs. In Table 15, the squares of the maximum components are displayed, indicating the states in resonance, together with the parameter $\delta_{-}$. Again, the correlation between the components and the LN parameter is manifested: going from local to normal mode behavior with the decrement in components manifested. This correlation is explicitly presented in Ref. [54].

## 8. Conclusions

In this contribution, we have presented, for the first time, a consistent set of LN criteria that can be applied to any molecule. In contrast to the long established theory of local molecules, where the criterion of locality depends on a model of interacting Morse oscillators, our criteria are based on the analysis of normal modes. Choosing a selected set of molecules, we have shown that the proposed LN criteria can be applied to a great variety of situations, from local to normal extremes. Each parameter presents its own features. The parameter $\delta_{-}$ measures the degree of locality from the normal point of view, while the Hessian $\gamma^{\left(\omega \right)}$ can be associated with a local perspective. In addition, $\epsilon_{1}$ (and in some cases $\epsilon_{2}$ too) offers a third parameter, and this is defined taking ingredients from both local and normal mode schemes through the force constants. Analyses of several representative systems were presented. First, the most simple system of two oscillators was presented, in order to include the case where the normal modes do not coincide with symmetry-adapted coordinates. The pyramidal molecules were included, in order to show that the proposed parameters can also be applied to the bending degrees of freedom. In addition, tetrahedral molecules were studied because of the presence of spurious modes in the bending modes. Finally, octahedral molecules were incorporated in our analysis because of the presence of two types of interactions involving vanishing contributions of the Wilson matrix. Although this set of studied systems may be considered relatively simple, it was chosen to include every possibility encountered in the framework of local coordinates, which allowed us to conclude the validity of our LN criteria.

The correlations between the different parameters in these systems were investigated to prove their consistency, but also to show that through such correlations it is possible to identify particular signatures of the molecules with just the knowledge of the fundamental energies:non-rigidity in NH${}_{3}$, resonance structures in O${}_{3}$ and NO${}_{2}$, and change in electronic configuration in the pnictogen pyramidal hydrides and GeH${}_{4}$. The isotopologues displayed a perfect linear correlation for the plots $\delta_{-}$ vs. $\gamma^{\left(\omega \right)}$. This fact allowed us to establish a linear correlation between the slopes and the force constants.

An important result is that the LN degree is correlated with physical properties. This conclusion was obtained by considering the behavior of the partition function for two oscillators, taking advantage of the parameterization H${}_{2}$O → CO${}_{2}$. A similar situation appears in spectroscopy; a clear dependence of the wave functions with the LN parameters, together with their correlation with the interaction strengths allowed us to conclude the importance of the LN parameters in the analysis of Raman and infrared spectroscopy. This finding is particularly relevant for series of isotopologues, where preliminary results indicated the existence of a correlation between the LN parameters and the relative transition intensities in the Raman spectra of the isotopologues of CO${}_{2}$.

The present formalism is based on a harmonic oscillator model, and only the fundamentals were involved in both the determination of the LN parameters and their correlation. Consequently, the proposed criteria can be applied to any molecular system, without limitations. A remarkable result is that these parameters are correlated with spectroscopic properties, taking into account the full description of the systems, where all the relevant interactions are included: anharmonicities and resonances. On the other hand, we selected internal coordinates in our treatment because of the physical meaning of the force constants, but in practice Cartesian coordinates are more appropriate to generalize our approach. In the latter case, efficient programs to obtain the normal modes are available and a work in this direction is in progress.

Finally, we conclude from this work that the LN parameters, in particular the $\delta_{-}$, is a descriptor of a molecule, which opens the possibility of also being used in machine learning algorithms.

## Figures and Tables

**Table 1:** Calculated force constants $f_{q_{i}q_{j}}^{\left(N\right)}$ using expressions (3) and $f_{q_{i}q_{j}}^{\left(L\right)}$ (11) for the different systems we have included in our analyses. Here, the $ћ\Omega_{\Gamma }$ corresponds to the fundamental energy of the Γ-th mode and *N* stands for the number of oscillators. $G_{\Gamma \Gamma }^{\left(0\right)}$ and $g_{qq}^{\left(0\right)}$ are the elements of the Wilson matrix in the normal and local coordinate basis, respectively. The meanings of $\mu_{2}$ and $\mu_{3}$ are irreducible representations and will be specified for each case along the text.

Force Constants from (3)	Force Constants from (11)
$f_{qq}^{\left(N\right)}=\frac{1}{N}\sum_{\Gamma }\frac{n_{\Gamma }\Omega_{\Gamma }^{2}}{G_{\Gamma \Gamma }^{\left(0\right)}}$	$f_{qq}^{\left(L\right)}=\frac{1}{g_{qq}^{\left(0\right)}}{\left(\frac{1}{N}\sum_{\Gamma }n_{\Gamma }\Omega_{\Gamma }\right)}^{2}$
$f_{qq^{\prime }}^{\left(N\right)}=\frac{1}{N}\sum_{\Gamma }{\left(-\right)}^{\delta_{\Gamma ,\mu_{2}}}\frac{\Omega_{\Gamma }^{2}}{G_{\Gamma \Gamma }^{\left(0\right)}}$	$f_{qq^{\prime }}^{\left(L\right)}=f_{qq}^{\left(L\right)}\left[2\frac{\sum_{\Gamma }{\left(-\right)}^{\delta_{\Gamma ,\mu_{2}}}\Omega_{\Gamma }}{\sum_{\Gamma }n_{\Gamma }\Omega_{\Gamma }}-x_{g}^{\prime }\right]$
$f_{qq^{\prime \prime }}^{\left(N\right)}=\frac{1}{N}\sum_{\Gamma }{\left(-\right)}^{\delta_{\Gamma ,\mu_{3}}}\frac{n_{\Gamma }\Omega_{\Gamma }^{2}}{G_{\Gamma \Gamma }^{\left(0\right)}}$	$f_{qq^{\prime \prime }}^{\left(L\right)}=f_{qq}^{\left(L\right)}\left[2\frac{\sum_{\Gamma }{\left(-\right)}^{\delta_{\Gamma ,\mu_{3}}}n_{\Gamma }\Omega_{\Gamma }}{\sum_{\Gamma }n_{\Gamma }\Omega_{\Gamma }}-x_{g}^{\prime \prime }\right]$

**Table 2:** Fundamentals and force constants calculated using the expressions in Table 1. References from which the fundamental energies were taken are indicated.

Molecule	$\nu_{1}$	$\nu_{3}$	$f_{rr}^{\left(N\right)}$	$f_{{rr}^{\prime }}^{\left(N\right)}$
H${}_{2}$O [43]	3657.053	3755.930	7.6756	−0.0910
H${}_{2}$S [44]	2614.408	2628.455	3.9569	−0.0168
H${}_{2}$Se [45]	2344.36	2357.65	3.2413	−0.0177
SO${}_{2}$ [46]	1151.71	1362.06	10.0004	−0.0233
F${}_{2}$O [47]	928.07	831	4.0864	0.9397
Cl${}_{2}$O [48]	641.97	686.54	3.0032	0.5452
O${}_{3}$ [49]	1103.14	1042.08	5.7941	1.6114
NO${}_{2}$ [50]	1319.79	1616.85	10.2889	1.8984
CO${}_{2}$ [51]	1285.41	2349.14	14.8871	0.6891
CS${}_{2}$ [52]	658.00	1535.35	7.6021	0.5767

**Table 3:** LN parameters for the molecules given in Table 2. Parenthesis (x) means $\times 10^{x}$.

Molecule	$\delta_{-}$	$\gamma^{\left(\omega \right)}$	ζ	$\epsilon_{1}$	$\epsilon_{2}$
H${}_{2}$O	5.47 (−7)	1.09 (−6)	0.0170	2.19 (−6)	−2.73 (−6)
H${}_{2}$S	6.06 (−7)	1.21 (−6)	0.0034	2.42 (−6)	−6.42 (−7)
H${}_{2}$Se	1.73 (−6)	3.46 (−6)	0.0036	6.92 (−6)	−2.51 (−7)
SO${}_{2}$	1.68 (−3)	3.26 (−3)	0.1056	6.57 (−3)	−0.4635
F${}_{2}$O	8.05 (−3)	0.0156	0.0700	0.0312	0.0167
Cl${}_{2}$O	0.0118	0.0228	0.0426	0.0457	0.0618
O${}_{3}$	0.0167	0.0317	0.0358	0.0634	0.0514
NO${}_{2}$	0.0211	0.0386	0.1271	0.0779	0.1567
CO${}_{2}$	0.0331	0.0477	0.3371	0.1033	1.2741
CS${}_{2}$	0.0736	0.0807	0.4296	0.1855	1.7789

**Table 4:** Fundamentals (in cm${}^{-1}$) and calculated force constants (in aJ Å${}^{-2}$) for certain pyramidal molecules. References from which the fundamental energies were taken are indicated.

Molecule	$\nu_{1}$	$\nu_{2}$	$\nu_{3}$	$\nu_{4}$	$f_{\mathrm{rr}}^{\left(N\right)}$	$f_{{\mathrm{rr}}^{\prime }}^{\left(N\right)}$	$f_{\theta \theta }^{\left(N\right)}$	$f_{\theta \theta^{\prime }}^{\left(N\right)}$
${}^{14}$NH${}_{3}$ [62]	3336.08	932.43	3443.68	1626.28	6.3871	−0.0117	0.5252	−0.0815
${}^{14}$NT${}_{3}$ [63]	2014.1	656.37	2184.76	996.28	6.5989	−0.0138	0.5402	−0.0645
${}^{15}$NCl${}_{3}$ [64]	541.7	364.8	632.3	262.8	2.4275	0.3837	0.2321	0.0571
${}^{14}$NCl${}_{3}$ [64]	554.2	365.2	644	263	2.4189	0.4073	0.2202	0.0533
${}^{31}$PH${}_{3}$ [65]	2321.12	992.13	2326.87	1118.31	3.0853	0.0006	0.3303	−0.0203
PCl${}_{3}$ [66]	515	258.3	504	186	2.5695	0.3104	0.1963	0.0222
PBr${}_{3}$ [66]	390	159.9	384.4	112.8	2.0586	0.3502	0.0959	0.0072
${}^{75}$AsH${}_{3}$ [67]	2115.16	906.75	2126.42	999.23	2.6198	−0.0080	0.2727	−0.0139
AsF${}_{3}$ [68]	740	338	703	263	4.5824	0.2579	0.3644	0.0489
AsCl${}_{3}$ [66]	416.5	192.5	319	150.2	2.2860	0.2180	0.1829	0.0211
AsBr${}_{3}$ [66]	272	128	287	99	1.8326	0.1105	0.1236	0.0079
${}^{121}$SbH${}_{3}$ [69]	1890.50	782.25	1894.50	827.86	2.0944	−0.0025	0.1929	−0.0053
SbF${}_{3}$ [70]	666	250	634	213	4.0305	0.1828	0.2436	0.0223
SbCl${}_{3}$ [66]	380.7	150.8	358.9	121.8	2.1794	0.1529	0.1364	0.0156
BiH${}_{3}$ [71]	1733.25	726.7	1734.47	751.24	1.7632	−0.0008	0.1619	−0.0035
BiCl${}_{3}$ [66]	342	123	322	107	1.9351	0.1179	0.1112	0.0116
BiBr${}_{3}$ [66]	220	77	214	63	1.5975	0.0998	0.0750	0.0076
BiI${}_{3}$ [66]	162	59.7	163.5	47	1.2453	0.0747	0.0575	0.0056

**Table 5:** Parameters $\delta_{-},\gamma^{\left(\omega \right)},\zeta ,\epsilon_{i}$ associated with the stretching degrees of freedom for several pyramidal molecules. Parenthesis (x) means $\times 10^{x}$.

Molecule	$\delta_{-}$	$\gamma^{\left(\omega \right)}$	ζ	$\epsilon_{1}$	$\epsilon_{2}$
${}^{14}$NT${}_{3}$	4.00 (−4)	5.91 (−4)	0.0516	0.0012	−0.3214
SbF${}_{3}$	4.84 (−4)	8.09 (−4)	0.0313	0.0016	0.0180
AsF${}_{3}$	8.79 (−4)	0.0015	0.0326	0.0029	0.0268
BiBr${}_{3}$	0.0016	0.0027	0.0176	0.0053	0.0463
BiI${}_{3}$	0.0024	0.0037	0.0059	0.0075	0.0703
AsBr${}_{3}$	0.0037	0.0056	0.0341	0.0114	0.1118
PCl${}_{3}$	0.0072	0.0117	0.0137	0.0231	0.1138
PBr${}_{3}$	0.0150	0.0237	0.0092	0.0468	0.1755
${}^{15}$NCl${}_{3}$	0.0264	0.0348	0.0975	0.0726	0.3282
${}^{14}$NCl${}_{3}$	0.0284	0.0375	0.0947	0.0782	0.3339

**Table 6:** LN parameters $\delta_{-},\gamma^{\left(\omega \right)},\zeta ,\epsilon_{i}$ for the bending degrees of freedom of pyramidal molecules. Parenthesis (x) means $\times 10^{x}$.

Molecule	$\delta_{-}$	$\gamma^{\left(\omega \right)}$	ζ	$\epsilon_{1}$	$\epsilon_{2}$
${}^{121}$SbH${}_{3}$	5.09 (−5)	7.70 (−5)	0.0360	1.57 (−4)	−0.0029
BiH${}_{3}$	7.12 (−5)	1.10 (−4)	0.0211	2.23 (−4)	−0.0052
${}^{14}$NH${}_{3}$	1.33 (−4)	1.09 (−4)	0.3164	2.81 (−4)	−0.0012
${}^{75}$AsH${}_{3}$	2.60 (−4)	3.78 (−4)	0.0616	0.0008	−0.0078
${}^{31}$PH${}_{3}$	3.64 (−4)	5.16 (−4)	0.0758	0.0011	−0.0090
PCl${}_{3}$	1.29 (−6)	2.64 (−6)	0.2003	4.85 (−6)	1.64 (−5)
AsCl${}_{3}$	4.40 (−4)	8.56 (−4)	0.1541	0.0016	0.0061
SbCl${}_{3}$	8.42 (−4)	0.0016	0.1335	0.0030	0.0123
BiCl${}_{3}$	0.0017	0.0031	0.0880	0.0059	0.0286
${}^{14}$NCl${}_{3}$	0.0077	0.0153	0.2003	0.0281	0.0586

**Table 7:** Fundamental energies for several tetrahedral molecules, together with the force constants calculated in accordance with Table 1. References from which the fundamental energies were taken are indicated.

Molecule	$\nu_{1}$	$\nu_{2}$	$\nu_{3}$	$\nu_{4}$	$f_{\mathrm{rr}}^{\left(N\right)}$	$f_{{\mathrm{rr}}^{\prime }}^{\left(N\right)}$	$f_{\theta \theta }^{\left(N\right)}$	$f_{\theta \theta^{\prime }}^{\left(N\right)}$	$f_{\theta \theta^{\prime \prime }}^{\left(N\right)}$
${}^{12}$CH${}_{4}$ [72]	2916.482	1533.336	3019.493	1310.762	4.9144	0.0455	0.3424	−0.0776	−0.0321
${}^{13}$CH${}_{4}$ [72]	-	1533.493	-	1302.781	-	-	0.3413	−0.0776	−0.0310
${}^{12}$CD${}_{4}$ [72]	-	1091.652	-	997.871	-	-	0.3576	−0.0786	−0.0433
${}^{13}$CD${}_{4}$ [72]	-	1091.801	-	989.250	-	-	0.3565	−0.0786	−0.0422
${[}^{14}$NH${}_{4}{]}^{+}$ [66]	3040	1680	3145	1400	5.3914	0.0322	0.4024	−0.0931	−0.0300
${[}^{15}$NH${}_{4}{]}^{+}$ [66]	-	1646	-	1399	-	-	0.3957	−0.0894	−0.0381
[ND${}_{4}{]}^{+}$ [66]	-	1215	-	1065	-	-	0.4281	−0.0973	−0.0387
[NT${}_{4}{]}^{+}$ [66]	-	976	-	913	-	-	0.4290	−0.0940	−0.0528
${}^{28}$SiH${}_{4}$ [73,74]	2186.8723	970.93445	2189.1895	913.46879	2.7466	0.0311	0.1576	−0.0311	−0.0332
${}^{29}$SiH${}_{4}$ [74]	-	970.94842	-	912.18312	-	-	0.1575	−0.0311	−0.0331
${}^{30}$SiH${}_{4}$ [74]	-	970.96148	-	910.97961	-	-	0.1574	−0.0311	−0.0329
${}^{29}$SiD${}_{4}$ [75]	-	689.88679	-	672.93384	-	-	0.1628	−0.0314	−0.0373
${}^{30}$SiD${}_{4}$ [75]	-	689.89950	-	671.43227	-	-	0.1626	−0.0314	−0.0371
${}^{70}$GeH${}_{4}$ [76]	-	929.90124	-	821.54462	-	-	0.1360	−0.0285	−0.0219
${}^{72}$GeH${}_{4}$ [76]	-	929.90513	-	821.11703	-	-	0.1360	−0.0285	−0.0218
${}^{73}$GeH${}_{4}$ [76]	-	929.90728	-	820.91126	-	-	0.1359	−0.0285	−0.0218
${}^{74}$GeH${}_{4}$ [76,77]	2110.70051	929.90910	2111.14192	820.71165	2.6109	0.0115	0.1359	−0.0285	−0.0218
${}^{76}$GeH${}_{4}$ [76]	-	929.91308	-	820.32666	-	-	0.1359	−0.0285	−0.0217
CF${}_{4}$ [66]	908.4	-	1283.0	-	6.7545	0.8276	-	-	-
CCl${}_{4}$ [66]	460	-	792.765	-	3.1000	0.4401	-	-	-
CBr${}_{4}$ [66]	267	-	672	-	2.4546	0.3006	-	-	-
CI${}_{4}$ [66]	178	-	555	-	1.7372	0.2106	-	-	-
HfCl${}_{4}$ [66]	-	101.5	-	112	-	-	0.0672	−0.0120	−0.0193
[PH${}_{4}{]}^{+}$ [66]	-	1086	-	974	-	-	0.1867	−0.0389	−0.0311
[AsH${}_{4}{]}^{+}$ [66]	-	1024	-	941	-	-	0.1729	−0.0346	−0.0345

**Table 8:** LN parameters associated with the stretching degrees of freedom of tetrahedral molecules.

Molecule	$\delta_{-}$	γ	ζ	$\epsilon_{1}$	$\epsilon_{2}$
${}^{74}$GeH${}_{4}$	2.48 (−5)	7.92 (−5)	0.0001	5.94 (−5)	0.0090
${}^{28}$SiH${}_{4}$	1.64 (−4)	5.25 (−4)	0.0007	3.94 (−4)	0.0236
${[}^{14}$NH${}_{4}{]}^{+}$	2.63 (−4)	8.03 (−4)	0.0216	6.13 (−4)	0.0708
${}^{12}$CH${}_{4}$	4.03 (−4)	1.23 (−3)	0.0221	9.40 (−4)	0.0702
CF${}_{4}$	0.0545	0.1068	0.2097	0.0920	0.6536
CCl${}_{4}$	0.1038	0.1528	0.3109	0.1414	0.9123
CBr${}_{4}$	0.1976	0.1699	0.4531	0.1835	1.4336
CI${}_{4}$	0.2828	0.1813	0.5090	0.2099	1.6816

**Table 9:** LN parameters associated with the bending degrees of freedom of tetrahedral molecules.

Molecule	$\delta_{-}$	$\gamma^{\left(\omega \right)}$	$\gamma^{\left(1\right)}$	$\gamma^{\left(2\right)}$	ζ	$\epsilon_{1}$	$\epsilon_{2}$	$\epsilon_{3}$
${}^{13}$CH${}_{4}$	4.96 (−7)	6.05 (−7)	1.82 (−7)	1.21 (−7)	0.1027	−0.0655	9.40 (−4)	−0.7303
${}^{12}$CH${}_{4}$	3.00 (−6)	3.64 (−6)	1.09 (−6)	7.28 (−7)	0.0988	−0.0611	6.87 (−4)	−0.6576
${[}^{14}$NH${}_{4}{]}^{+}$	1.10 (−5)	1.34 (−5)	4.03 (−6)	2.69 (−6)	0.1145	−0.0688	7.02 (−4)	−0.9332
${[}^{15}$NH${}_{4}{]}^{+}$	1.29 (−5)	1.57 (−5)	4.71 (−6)	3.14 (−6)	0.1024	−0.0667	1.43 (−3)	−0.7056
${}^{13}$CD${}_{4}$	1.87 (−4)	2.19 (−4)	6.56 (−5)	4.38 (−5)	0.0625	−0.0168	5.00 (−4)	−0.1460
${}^{12}$CD${}_{4}$	2.56 (−4)	2.98 (−4)	8.94 (−5)	5.96 (−5)	0.0570	−0.0094	9.76 (−4)	−0.0849
[ND${}_{4}{]}^{+}$	3.25 (−4)	3.87 (−4)	1.16 (−4)	7.74 (−5)	0.0833	−0.0223	8.30 (−4)	−0.2548
HfCl${}_{4}$	4.65 (−4)	4.81 (−4)	1.44 (−4)	9.63 (−5)	0.0624	7.69 (−3)	2.46 (−3)	0.0206
[NT${}_{4}{]}^{+}$	6.85 (−4)	7.80 (−4)	2.34 (−4)	1.56 (−4)	0.0424	0.0164	4.42 (−3)	0.1018
[PH${}_{4}{]}^{+}$	1.01 (−3)	1.22 (−3)	3.65 (−4)	2.44 (−4)	0.0690	−0.0986	0.0153	−0.6693
${}^{70}$GeH${}_{4}$	1.30 (−3)	1.58 (−3)	4.73 (−4)	3.16 (−4)	0.0784	−0.1127	0.0198	−0.8034
${}^{72}$GeH${}_{4}$	1.30 (−3)	1.58 (−3)	4.75 (−4)	3.16 (−4)	0.0787	−0.1130	0.0199	−0.8073
${}^{73}$GeH${}_{4}$	1.30 (−3)	1.58 (−3)	4.75 (−4)	3.17 (−4)	0.0789	−0.1132	0.0199	−0.8093
${}^{74}$GeH${}_{4}$	1.31 (−3)	1.59 (−3)	4.76 (−4)	3.17 (−4)	0.0790	−0.1133	0.0199	−0.8111
${}^{76}$GeH${}_{4}$	1.31 (−3)	1.59 (−3)	4.77 (−4)	3.18 (−4)	0.0793	−0.1136	0.0200	−0.8147
${}^{29}$GeD${}_{4}$	1.41 (−3)	1.63 (−3)	4.88 (−4)	3.25 (−4)	0.0158	−0.0701	0.0162	−0.3603
${}^{30}$GeD${}_{4}$	1.44 (−3)	1.66 (−3)	4.98 (−4)	3.32 (−4)	0.0173	−0.0717	0.0166	−0.3705
${}^{28}$GeH${}_{4}$	1.80 (−3)	2.13 (−3)	6.38 (−4)	4.25 (−4)	0.0388	−0.0945	0.0226	−0.5329
${}^{29}$GeH${}_{4}$	1.81 (−3)	2.15 (−3)	6.44 (−4)	4.29 (−4)	0.0397	−0.0954	0.0229	−0.5403
${}^{30}$GeH${}_{4}$	1.83 (−3)	2.16 (−3)	6.49 (−4)	4.33 (−4)	0.0405	−0.0963	0.0231	−0.5474
[AsH${}_{4}{]}^{+}$	2.16 (−3)	2.59 (−3)	7.76 (−4)	5.17 (−4)	0.0537	−0.1121	0.0283	−0.6754

**Table 10:** Fundamentals (in ${\mathrm{cm}}^{-1}$) from Ref. [66] and force constants (in aJ ${Å}^{-2}$) for octahedral molecules.

Molecule	$\nu_{1}$	$\nu_{2}$	$\nu_{3}$	$f_{\mathrm{rr}}^{\left(N\right)}$	$f_{{\mathrm{rr}}^{\prime }}^{\left(N\right)}$	$f_{{\mathrm{rr}}^{\prime \prime }}^{\left(N\right)}$
PuF_6_	625	519	612	3.5476	0.2262	−0.0801
NpF_6_	646	525	618	3.6491	0.2643	−0.0352
[PtF_6_${]}^{2-}$	611	576	571	3.4617	0.0775	0.4071
[PbCl_6_${]}^{2-}$	281	209	262	1.1132	0.1228	0.0449
[HfCl_6_${]}^{2-}$	325	257	275	1.3928	0.1378	0.2623
[GaF_6_${]}^{3-}$	535	398	481	1.9631	0.2385	0.2869
[SbBr_6_${]}^{-}$	192	169	239	1.3189	0.0651	0.1560
[SiF_6_${]}^{2-}$	663	477	741	2.9751	0.3956	0.3629
[AlF_6_${]}^{3-}$	541	400	568	1.8928	0.2475	0.3932
[TiCl_6_${]}^{2-}$	320	271	316	1.2881	0.1008	0.4475

**Table 11:** LN parameters associated with the stretching degrees of freedom of octahedral molecules.

Molecule	$\delta_{-}$	$\gamma^{\left(\omega \right)}$	$\gamma^{\left(1\right)}$	$\gamma^{\left(2\right)}$	ζ	$\epsilon_{1}$	$\epsilon_{2}$	$\epsilon_{3}$
PuF_6_	0.0019	0.0023	0.0036	0.0041	0.1167	0.0045	0.0541	−0.1762
NpF_6_	0.0024	0.0031	0.0050	0.0052	0.1297	0.0059	0.0645	−0.5237
[PtF_6_${]}^{2-}$	0.0031	0.0056	0.0026	0.0005	0.0430	0.0110	0.1105	0.0117
[PbCl_6_${]}^{2-}$	0.0071	0.0104	0.0164	0.0122	0.1820	0.0185	0.1374	0.2959
[HfCl_6_${]}^{2-}$	0.0120	0.0206	0.0224	0.0098	0.1461	0.0377	0.2049	0.0673
[GaF_6_${]}^{3-}$	0.0142	0.0236	0.0293	0.0148	0.1818	0.0422	0.2212	0.1258
[SbBr_6_${]}^{-}$	0.0230	0.0343	0.0140	0.0024	0.2104	0.0691	0.3048	0.2421
[SiF_6_${]}^{2-}$	0.0295	0.0434	0.0438	0.0177	0.2604	0.0807	0.3303	0.3378
[AlF_6_${]}^{3-}$	0.0368	0.0567	0.0492	0.0171	0.2127	0.1062	0.3670	0.2481
[TiCl_6_${]}^{2-}$	0.0481	0.0777	0.0333	0.0061	0.1046	0.1522	0.4135	0.1936

**Table 12:** Maximum components of the wave function compared with $\delta_{-}$ for the pyramidal molecules PH${}_{3}$, AsH${}_{3}$, and SbH${}_{3}$, taken from Ref. [20].

$\delta_{-}\times 10^{-6}$	0.143	0.685	1.042
State	SbH_3_	PH_3_	AsH_3_
|200000〉​*	0.989	0.968	0.951
|400000〉​*	0.981	0.627	0.555
|200100〉	0.467	0.756	0.722

**Table 13:** Transition involved in the most intense lines in the Raman spectrum. Labels $\Sigma^{+}\left(\Pi^{\pm }\right)$ and $\Sigma_{g}^{+}\left(\Pi_{u}^{\pm }\right)$ for the asymmetric and symmetric isotopologues, respectively. Given the normalization, the parameters *a* and $a^{\prime }$ determine the eigenstates and the transition strength.

Symmetry	$\left|\nu \rangle \to \right|\nu^{\prime }\rangle$
$\Sigma^{+},\Sigma_{g}^{+}$	$|00^{0}0\rangle \to a|10^{0}0\rangle +b|02^{0}0\rangle$
$\Sigma^{+},\Sigma_{g}^{+}$	$|00^{0}0\rangle \to b|10^{0}0\rangle -a|02^{0}0\rangle$
$\Pi^{\pm },\Pi_{u}^{\pm }$	$|01^{\pm 1}0\rangle \to a^{\prime }|11^{\pm 1}0\rangle +b^{\prime }|03^{\pm 1}0\rangle$
$\Pi^{\pm },\Pi_{u}^{\pm }$	$|01^{\pm 1}0\rangle \to b^{\prime }|11^{\pm 1}0\rangle -a^{\prime }|03^{\pm 1}0\rangle$

**Table 14:** Components of the wave functions, as well as the parameter $\delta_{-}$. The notation for the isotopologues is ${}^{1x}$O${}^{1y}$C${}^{1z}$O →(xyz).

	(636)	(637)	(638)	(626)	(738)	(627)	(628)	(727)	(728)	(828)
*a*	0.5587	0.604859	0.645312	0.6983	0.747715	0.75071	0.794356	0.7826	0.830245	0.8488
$a^{\prime }$	0.6076	0.636241	0.656285	0.7045	0.722074	0.743765	0.778424	0.7586	0.805748	0.8150
$\delta_{-}$	0.03375	0.03487	0.03594	0.3684	0.03717	0.038035	0.03915	0.03933	0.04051	0.04181

**Table 15:** Square of the maximum components for the wave functions involved in the multiplets (84). The components were taken from Ref. [54].

	$\delta_{-}$	0.02626	0.02894	0.03035
Polyad	States	(934)	(924)	(925)
2	$|02^{0}0\rangle$–$|10^{0}0\rangle$	0.961	0.944	0.937
4	$|04^{0}0\rangle$–$|12^{0}0\rangle$	0.875	0.834	0.816
4	$|20^{0}0\rangle$–$|00^{0}1\rangle$	0.844	0.827	0.811
3	$|03^{1}0\rangle$–$|11^{1}0\rangle$	0.930	0.904	0.890
5	$|05^{1}0\rangle$–$|13^{1}0\rangle$	0.820	0.781	0.750
5	$|21^{1}0\rangle$–$|01^{1}1\rangle$	0.756	0.735	0.712

## Data Availability

The original contributions presented in the study are included in the article, further inquiries can be directed to the corresponding author.

## References

[1] Herzberg G. (1945). Molecular Spectra and Molecular Structure II. Infrared and Raman Spectra.

[2] Wilson E.B., Decious J.C., Cross P.C. (1955). Molecular Vibrations. The Theory of Infrared and Raman Spectra.

[3] Fermi E. (1931). Über den Ramaneffekt des Kohlendioxyds. Z. FüR Phys..

[4] Darling B.T., Dennison D.M. (1940). The Water Vapor Molecule. Phys. Rev..

[5] Hollas J.M. (1982). High Resolution Spectroscopy.

[6] Walther H. (1976). Laser Spectroscopy of Atoms and Molecules.

[7] Quack M. (1990). Spectra and Dynamics of Coupled Vibrations in Polyatomic Molecules. Annu. Rev. Phys. Chem..

[8] Mecke R. (1932). Valenz-und Deformationsschwingungen mehratomiger Moleküle. III. Z. FüR Phys. Chem..

[9] Mecke R. (1933). Das Rotationsschwingungsspektrum des Wasserdampfes. I. Z. FüR Phys..

[10] Timm B., Mecke R. (1935). Quantitative Absorptionsmessungen an den CH-Oberschwingungen einfacher Kohlenwasserstoffe. Z. FüR Phys..

[11] Rumpf K., Mecke R. (1939). Über die Absorptionsspektren einiger Benzolderivate im nahen Ultraroten bei großen Schichtdicken. Z. FüR Phys. Chem..

[12] Halonen L., Child M.S. (1983). Model stretching overtone eigenvalues for SF_6_, WF_6_, and UF_6_. J. Chem. Phys..

[13] Child M.S., Halonen L. (1984). Overtone Frequencies and Intensities in the Local Mode Picture.

[14] Halonen L. (1998). Local Mode Vibrations in Polyatomic Molecules.

[15] Halonen L. (2000). Highly Excited States and Local Modes.

[16] Jensen P. (2000). An introduction to the theory of local mode vibrations. Mol. Phys..

[17] Child M.S., Lawton R.T. (1981). Local and normal vibrational states: A harmonically coupled anharmonic-oscillator model. Faraday Discuss. Chem. Soc..

[18] Mills I., Robiette A. (1985). On the relationship of normal modes to local modes in molecular vibrations. Mol. Phys..

[19] Halonen L. (1997). Internal coordinate Hamiltonian model for Fermi resonances and local modes in methane. J. Chem. Phys..

[20] Guzmán-Juárez O., Suárez E., Lemus R. (2023). A study of the local to normal mode transition in pyramidal molecules and their vibrational description in terms of an algebraic model. J. Mol. Spectrosc..

[21] Sibert E.L., Hynes J.T., Reinhardt W.P. (1982). Quantum mechanics of local mode ABA triatomic molecules. J. Chem. Phys..

[22] Davis M.J. (1995). Analysis of highly excited vibrational eigenstates. Int. Rev. Phys. Chem..

[23] Heller E.J., Davis M.J. (1980). Molecular overtone bandwidths from classical trajectories. J. Phys. Chem..

[24] Kellman M.E., Tyng V. (2007). The Dance of Molecules: New Dynamical Perspectives on Highly Excited Molecular Vibrations. Accounts Chem. Res..

[25] Xiao L., Kellman M.E. (1989). Unified semiclassical dynamics for molecular resonance spectra. J. Chem. Phys..

[26] Schmid G., Coy S., Field R., Silbey R. (1994). Duffing’s oscillator and the normal to local mode transition in AB_2_ triatomic molecules. Chem. Phys. Lett..

[27] Kellman M.E., Lynch E.D. (1988). Semiclassical phase space evolution of Fermi resonance spectra. J. Chem. Phys..

[28] Sako T., Yamanouchi K., Iachello F. (2001). Classical-limit analysis of the normal-to-local transition by the algebraic effective Hamiltonian approach. J. Chem. Phys..

[29] Xiao L., Kellman M.E. (1990). Catastrophe map classification of the generalized normal–local transition in Fermi resonance spectra. J. Chem. Phys..

[30] Bermúdez-Montaña M., Lemus R., Castaños O. (2016). Fidelity, entropy, and Poincaré sections as tools to study the polyad breaking phenomenon. EPL (Europhys. Lett.).

[31] Bermúdez-Montaña M., Lemus R., Castaños O. (2017). Polyad breaking phenomenon associated with a local-to-normal mode transition and suitability to estimate force constants. Mol. Phys..

[32] Coello L., Lemus R. (2021). Vibrational description of the stretching modes of octahedral molecules: A local-to-normal mode transition criterion. Mol. Phys..

[33] Bermúdez-Montaña M., Rodríguez-Arcos M., Carvajal M., Ostertag-Henning C., Lemus R. (2023). A Spectroscopic Description of Asymmetric Isotopologues of CO_2_. J. Phys. Chem. A.

[34] Bunker P.R., Jensen P. (1998). Molecular Synnetry and Spectroscopy.

[35] Mills I., Mompean F. (1986). Local modes and *x*, *k* relations in ethene and propadiene. Chem. Phys. Lett..

[36] Valle R.G.D. (1988). Local-mode to normal-mode hamiltonian transformation for X − H stretchings. Mol. Phys..

[37] Lehmann K.K. (1989). Beyond the *x* − *K* relations. Mol. Phys..

[38] Law M.M., Duncan J.L. (1998). Anharmonic stretching vibrations expressed as local modes. Mol. Phys..

[39] Law M.M., Duncan J.L. (1998). Anharmonically-coupled local mode to normal mode Hamiltonian transformations: Beyond the *x*, *K* relations. Mol. Phys..

[40] Gambi A., Valle R.G.D. (2007). Local mode and normal mode models for molecules with two non-equivalent C–H bonds. Mol. Phys..

[41] Lehmann K.K. (1983). On the relation of Child and Lawton’s harmonically coupled anharmonic–oscillator model and Darling–Dennison coupling. J. Chem. Phys..

[42] Kellman M.E. (1985). Algebraic resonance dynamics of the normal/local transition from experimental spectra of ABA triatomics. J. Chem. Phys..

[43] Kjaergaard H.G., Henry B.R., Wei H., Lefebvre S., Carrington T., Mortensen O.S., Sage M.L. (1994). Calculation of vibrational fundamental and overtone band intensities of H_2_O. J. Chem. Phys..

[44] Naumenko O., Campargue A. (2001). H_2_^32^S: First Observation of the (70^±^,0) Local Mode Pair and Updated Global Effective Vibrational Hamiltonian. J. Mol. Spectrosc..

[45] Flaud J., Camypeyret C., Burger H., Willner H. (1993). High-Resolution Analysis of the *ν*_2_, 2*ν*_2_, *ν*_1_, and *ν*_3_ Bands of $H_{2}^{80}$Se. J. Mol. Spectrosc..

[46] Kauppi E., Halonen L. (1992). A simple curvilinear internal coordinate model for vibrational energy levels. J. Chem. Phys..

[47] Saarinen T., Kauppi E., Halonen L. (1990). Potential energy surface for F_2_O. J. Mol. Spectrosc..

[48] Aures R., Gericke K.H., Maul C., Trott-Kriegeskorte G., Kawasaki M., Nakano Y. (2002). Excited state dynamics of Cl_2_O in the near ultraviolet. J. Chem. Phys..

[49] Babikov Y.L., Mikhailenko S.N., Barbe A., Tyuterev V.G. (2014). S&MPO—An information system for ozone spectroscopy on the WEB. J. Quant. Spectrosc. Radiat. Transf..

[50] Delon A., Jost R. (1991). Laser induced dispersed fluorescence spectra of jet cooled NO_2_: The complete set of vibrational levels up to 10000 cm^−1^ and the onset of the ${\tilde{X}}^{2}$*A*_1_ − ${\tilde{A}}^{2}$*B*_2_ vibronic interaction. J. Chem. Phys..

[51] Miller C.E., Brown L.R. (2004). Near infrared spectroscopy of carbon dioxide I. ^16^O^12^C^16^O line positions. J. Mol. Spectrosc..

[52] Suzuki I. (1975). General Anharmonic Force Constants of Carbon Disulfide. Bull. Chem. Soc. Jpn..

[53] Bermúdez-Montaña M., Rodríguez-Arcos M., Carvajal M., Ostertag-Henning C., Lemus R. (2022). Algebraic vibrational description of the symmetric isotopologues of CO_2_: ^13^C^16^O_2_, ^12^C^18^O_2_, ^12^C^17^O_2_. Chem. Phys..

[54] Suárez E., Guzmán-Juárez O., Lemus R. A general local algebraic approach for molecules with normal mode behavior: Application to FCN.

[55] Tennyson J., Bernath P.F., Brown L.R., Campargue A., Carleer M.R., Császár A.G., Gamache R.R., Hodges J.T., Jenouvrier A., Naumenko O.V. (2009). IUPAC critical evaluation of the rotational–vibrational spectra of water vapor. Part I—Energy levels and transition wavenumbers for $H_{2}^{17}$O and $H_{2}^{18}$O. J. Quant. Spectrosc. Radiat. Transf..

[56] Tennyson J., Bernath P.F., Brown L.R., Campargue A., Császár A.G., Daumont L., Gamache R.R., Hodges J.T., Naumenko O.V., Polyansky O.L. (2013). IUPAC critical evaluation of the rotational–vibrational spectra of water vapor, Part III: Energy levels and transition wavenumbers for $H_{2}^{16}$O. J. Quant. Spectrosc. Radiat. Transf..

[57] Tennyson J., Bernath P.F., Brown L.R., Campargue A., Császár A.G., Daumont L., Gamache R.R., Hodges J.T., Naumenko O.V., Polyansky O.L. (2014). IUPAC critical evaluation of the rotational–vibrational spectra of water vapor. Part IV. Energy levels and transition wavenumbers for $D_{2}^{16}$O, $D_{2}^{17}$O, and $D_{2}^{18}$O. J. Quant. Spectrosc. Radiat. Transf..

[58] Tóbiás R., Furtenbacher T., Császár A.G., Naumenko O.V., Tennyson J., Flaud J.M., Kumar P., Poirier B. (2018). Critical evaluation of measured rotational–vibrational transitions of four sulphur isotopologues of S^16^O_2_. J. Quant. Spectrosc. Radiat. Transf..

[59] Perrin A., Flaud J.M., Camy-Peyret C., Vasserot A.M., Guelachvili G., Goldman A., Murcray F., Blatherwick R. (1992). The *ν*_1_, 2*ν*_2_, and *ν*_3_ interacting bands of ^14^N^16^O_2_: Line positions and intensities. J. Mol. Spectrosc..

[60] Perrin A., Toon G., Orphal J. (2015). Detection of atmospheric ^15^NO_2_ in the *ν*_3_ spectral region (6.3 µm). J. Quant. Spectrosc. Radiat. Transf..

[61] Amezcua-Eccius C., Lemus R. (2010). An approach for the description of vibrational excitations: Application to ^11^BF_3_. J. Mol. Spectrosc..

[62] Rajamäki T., Miani A., Halonen L. (2003). Six-dimensional ab initio potential energy surfaces for H_3_O^+^ and NH_3_: Approaching the subwave number accuracy for the inversion splittings. J. Chem. Phys..

[63] Rajamäki T., Miani A., Halonen L. (2003). Vibrational energy levels for symmetric and asymmetric isotopomers of ammonia with an exact kinetic energy operator and new potential energy surfaces. J. Chem. Phys..

[64] Sawodny W., Härtner H., Minkwitz R., Bernstein D. (1989). Vibrational spectra and force constants for the molecular series NH_3−*n*_Cl*n*. J. Mol. Struct..

[65] Yurchenko S.N., Carvajal M., Thiel W., Jensen P. (2006). Ab initio dipole moment and theoretical rovibrational intensities in the electronic ground state of PH_3_. J. Mol. Spectrosc..

[66] Nakamoto K. (2009). Infrared and Raman Spectra of Inorganic Compounds. Part A: Theory and Applications in Inorganic Chemistry.

[67] Sánchez-Castellanos M., Álvarez Bajo O., Amezcua-Eccius C., Lemus R. (2006). Vibrational excitations of arsine in the framework of a local unitary group approach. J. Mol. Spectrosc..

[68] Bürger H., Ruland H., Demaison J., Dréan P. (2000). The *ν*_1_ = 1 and *ν*_3_ = 1 states of AsF_3_ studied by high resolution IR and millimeterwave spectroscopy: The equilibrium structure of AsF_3_. J. Mol. Struct..

[69] Fusina L., Lonardo G.D. (2002). The Fundamental Bands in the Infrared Spectrum of Stibine (SbH_3_). J. Mol. Spectrosc..

[70] Molnár J., Kolonits M., Hargittai M. (1997). Molecular structure of SbF_3_ and BiF_3_: An electron diffraction study. J. Mol. Struct..

[71] Jerzembeck W., Bürger H., Breidung J., Thiel W. (2004). High resolution infrared spectra of the *ν*_1_–*ν*_4_ bands of BiH_3_, and ab initio calculations of the spectroscopic parameters. J. Mol. Spectrosc..

[72] Boudon V., Rey M., Loëte M. (2006). The vibrational levels of methane obtained from analyses of high-resolution spectra. J. Quant. Spectrosc. Radiat. Transf..

[73] Ulenikov O., Gromova O., Bekhtereva E., Raspopova N., Fomchenko A., Sydow C., Bauerecker S. (2017). High resolution study of strongly interacting *ν*_3_(*F*_2_)/*ν*_1_(*A*_1_) bands of ^M^SiH_4_ (M = 28, 29, 30). J. Quant. Spectrosc. Radiat. Transf..

[74] Ulenikov O., Gromova O., Bekhtereva E., Raspopova N., Kashirina N., Fomchenko A., Sydow C., Bauerecker S. (2017). High resolution study of ^M^SiH_4_ (M = 28, 29, 30) in the Dyad Region: Analysis of line positions, intensities and half-widths. J. Quant. Spectrosc. Radiat. Transf..

[75] Sydow C., Gromova O., Bekhtereva E., Raspopova N., Belova A., Bauerecker S., Ulenikov O. (2019). First high–resolution analysis of the fundamental bands of ^29^SiD_4_ and ^30^SiD_4_: Line positions and strengths. J. Quant. Spectrosc. Radiat. Transf..

[76] Richard C., Boudon V., Rizopoulos A., Auwera J.V., Tchana F.K. (2021). Line positions and intensities for the *ν*_2_/*ν*_4_ bands of 5 isotopologues of germane near 11.5 μm. J. Quant. Spectrosc. Radiat. Transf..

[77] Boudon V., Grigoryan T., Philipot F., Richard C., Tchana F.K., Manceron L., Rizopoulos A., Auwera J.V., Encrenaz T. (2018). Line positions and intensities for the *ν*_3_ band of 5 isotopologues of germane for planetary applications. J. Quant. Spectrosc. Radiat. Transf..

[78] Bermúdez-Montaña M., Carvajal M., Pérez-Bernal F., Lemus R. (2020). An algebraic alternative for the accurate simulation of CO_2_ Raman spectra. J. Raman Spectrosc..

